# Nucleosome stability measured *in situ* by automated quantitative imaging

**DOI:** 10.1038/s41598-017-12608-9

**Published:** 2017-10-06

**Authors:** László Imre, Zoltán Simándi, Attila Horváth, György Fenyőfalvi, Péter Nánási, Erfaneh Firouzi Niaki, Éva Hegedüs, Zsolt Bacsó, Urbain Weyemi, Rebekka Mauser, Juan Ausio, Albert Jeltsch, William Bonner, László Nagy, Hiroshi Kimura, Gábor Szabó

**Affiliations:** 1 Department of Biophysics and Cell Biology, University of Debrecen, Debrecen, H-4032 Hungary; 2 Department of Biochemistry and Molecular Biology, University of Debrecen, Debrecen, H-4032 Hungary; 3 Center for Cancer Research National Cancer Institute, Bethesda, Maryland 20892 USA; 4 Cell Biology Unit, Institute of Innovative Research, Tokyo Institute of Technology, Yokohama, 226-8501 Japan; 5 Institute of Biochemistry, Stuttgart University, Stuttgart, Germany; 6 Sanford Burnham Prebys Medical Discovery Institute, Orlando, Florida USA; 7 University of Victoria, Department of Biochemistry, Victoria, BC V8W 3P6 Canada; 8 MTA-DE “Lendulet” Immunogenomics Research Group, University of Debrecen, Debrecen, Hungary

## Abstract

Current approaches have limitations in providing insight into the functional properties of particular nucleosomes in their native molecular environment. Here we describe a simple and powerful method involving elution of histones using intercalators or salt, to assess stability features dependent on DNA superhelicity and relying mainly on electrostatic interactions, respectively, and measurement of the fraction of histones remaining chromatin-bound in the individual nuclei using histone type- or posttranslational modification- (PTM-) specific antibodies and automated, quantitative imaging. The method has been validated in H3K4me3 ChIP-seq experiments, by the quantitative assessment of chromatin loop relaxation required for nucleosomal destabilization, and by comparative analyses of the intercalator and salt induced release from the nucleosomes of different histones. The accuracy of the assay allowed us to observe examples of strict association between nucleosome stability and PTMs across cell types, differentiation state and throughout the cell-cycle in close to native chromatin context, and resolve ambiguities regarding the destabilizing effect of H2A.X phosphorylation. The advantages of the *in situ* measuring scenario are demonstrated via the marked effect of DNA nicking on histone eviction that underscores the powerful potential of topological relaxation in the epigenetic regulation of DNA accessibility.

## Introduction

Nucleosome structure is, in general, repressive; hence, stability of nucleosomes is of regulatory importance in eukaryotes^1–5^. Formation of nucleosome free regions (NFRs) is a prerequisite for downstream steps of transcriptional activation; release of nucleosomes at these sites is regulated by coupled histone acetylation, histone chaperone, remodelling and topoisomerase activity^6–10^. During transcriptional elongation, nucleosomes are disrupted and reassembled in the wake of RNA Pol II. This is thought to proceed via the transient release of the H2A – H2B dimers to yield hexasomes^11–13^ concurrently with the transcriptionally-coupled over- and underwinding of DNA in front of and behind RNA polymerase, respectively^14–17^.

The techniques that have proven to provide the most informative data for the assessment of nucleosome stability include biochemical or biophysical measurements on isolated or reconstituted nucleosomes^2,18–26^, approaches based on metabolic labeling^27,28^, biochemical strategies embedded in genomics approaches^29–31^, single-molecule^32^ magnetic tweezer or FRET measurements^33–37^, proteomic analyses^27,28,38,39^ and microscopic studies using transfected histones fused with fluorescent^40–42^ and photo-activatable proteins^43,44^. The above methods assess dissociation of histones from the nucleosomes either in live cells where it occurs spontaneously, or when purified or reconstituted nucleosomes are exposed to different ionic environments, or by evoking changes of superhelicity with the help of mechanical torsion or intercalators. However, none of these methods can readily and rapidly address the stability of histones with a specific posttranslational modification, i.e. within a given chromatin context, *in situ*, also allowing assessment of the role of DNA topology. The biophysical techniques that rely on transfected constructs^40,41,43^ provide information on exogenous fusion products, carrying no posttranslational modifications (PTMs), in relatively few cells, limiting their physiological relevance and accuracy. PTM specific information is derived from biochemical or biophysical studies involving *in vitro* modified, isolated or reconstituted nucleosomes without cell-to-cell resolution^23,45^. Comparison of nucleosomes in different PTM context by genomic approaches is feasible, with considerable limitations, however, in throughput due to the demand on the availability of bioinformatic expertise and the expenses incurred. In addition, the average features of large cell populations are revealed in these approaches, and the potential advantages of the individual cell perspective cannot be exploited. The individual characteristics of the different methods are compared in Table 1 (see Discussion).

**Table 1:** Comparison of QINESIn with other methods available for the examination of molecular features related to nucleosome stability.

	QINESIn	Proteomic analyses Refs (A)	Assays on isolated/reconstituted nucleosomes Refs (B)	Genomics approaches Refs (C)	Plasmid derived tagged histones Refs (D)
Quantitative analysis of nucleosome stability	+	+	+	+	+
Histone PTM specificity	+	+	+ −	+	−
Histone variant specificity	+	+	+	+	+
Overall expression or modification levels assessed	+	+	−	+ −	−
Measurement targets endogenous histones	+	+	+	+	−
Measurement of nucleosome stability *in situ*	+	−	−	−	+
Analyses according to cell cycle phases, without synchronization*	+	−	−	−	−
Assessment of superhelicity effects in situ**	+	−	−	−	−
Detection of molecular interactions by X-linking***	+	−	−	−	+
High-throughput screening	+	−	−	−	−
Comparison of different cell types in mixed-cell experiments****	+	−	−	−	−
Analysis according to cell surface markers in mixed-cell experiments	+	−	−	−	−
Cell-by-cell analyses	+	−	−	−	+
Sensitivity to heterogeneity (e. g. gating for different expression levels)*****	+	−	−	−	+
Picturing genome-wide distribution	−	−	−	+	−

“+” indicates that the particular approach has proved to be applicable for the corresponding purposes listed in the left column. “+/−” indicates that the particular approach provides semiquantitative or not readily extractable information. The asterisks refer to the data demonstrating the QINESIn features: *Supplementary Fig. S12; **Fig. 4C–F; Supplementary Fig. S10A; ***Supplementary Fig. S13 A,B; ****Figs 2D, 3D, Supplementary Fig. S2; *****Supplementary Fig. S11A,B. References: (A):^27,28,38,39,43^, (B):^2,18–26,32–37,45^, (C):^29–31,46^, (D):^40–44^.

PTMs, including those on the histone tails and the ones localized to the core regions, are key players in the regulation of nucleosomal stability^5,46–48^, yet the mechanisms involved in this regulation are poorly understood. How histone variants affect nucleosome stability is also a crucial question to be resolved on the way toward understanding their role in regulation^49,50^. That negative superhelicity, an obligatory feature of nucleosomal structure, is exploited for gene regulation has been demonstrated (ref.^51^ and therein) and much has been learned about the role of topoisomerases in the maintenance of supercoiled state in the course of chromatin dynamics^16,17^, but an option for the direct and *in situ* assessment of these relationships could lead to a better understanding of these intertwined issues. The technique presented provides a sensitive measuring platform also for such studies.

The assay delivers histone type and PTM specific information on the stability features of nucleosomes consisting of native endogenous or ectopically expressed histones as well, *in situ*, in the individual nuclei of a cell population, and is amenable to high throughput studies. Agarose embedded cells are lysed and exposed to salt or to DNA intercalating agents and the remaining chromatin-bound histones are detected using specific antibodies and quantitative microscopy conveniently performed by laser scanning cytometry (LSC), hence the name coined for the method: Quantitative Imaging of Nuclei after Elution with Salt/Intercalators (QINESIn). Exposure to salt will primarily affect electrostatic histone-histone and histone-DNA interactions^34,52^, while DNA intercalators extend, unwind, and at higher intercalator concentrations overwind the DNA, constraining the histone-DNA contacts^43,53,54^. DNA intercalators also increase the DNA melting temperature^55^ and are expected to affect higher-order chromatin structure^56,57^. The proof of concept of the technique is demonstrated by reproducing published PTM-specific effects of doxorubicin intercalation^43,58^. Our method not only reproduces the results of the previous observations but confirms them quantitatively, using other intercalators: Ethidium bromide (EBr; long known for its nucleosome destabilizing properties^19^ and references therein), and SYBR Gold, a DNA intercalating dye of high fluorescence quantum yield. The role of superhelicity relaxation in nucleosome destabilization is elucidated measuring the winding of the superhelical DNA in nuclear halos and studying the effect of nicking treatments. An interplay between salt induced and intercalation related changes has been revealed and the differential sensitivity of two elution methods demonstrated. The quantitative nature of the assay allows us to observe consistent, PTM-specific differences across cell type and differentiation state and cell-cycle phase. To further demonstrate the utility of the approach in studying histone type- and PTM-specific effects, we compare the stability of canonical and H2A.X-containing nucleosomes before and following phosphorylation of this histone variant yielding γH2A.X^59^. The data obtained by QUINESIn help dissolve the controversies regarding the effect of H2A.X phosphorylation on nucleosome stability^60^.

The method described herein opens an exciting window of opportunity for addressing a wide spectrum of questions related to the regulation of nucleosome stability by PTMs, histone composition and DNA superhelicity.

## Results

### Nucleosome stability measured by doxorubicin elution

**Figure 1:**
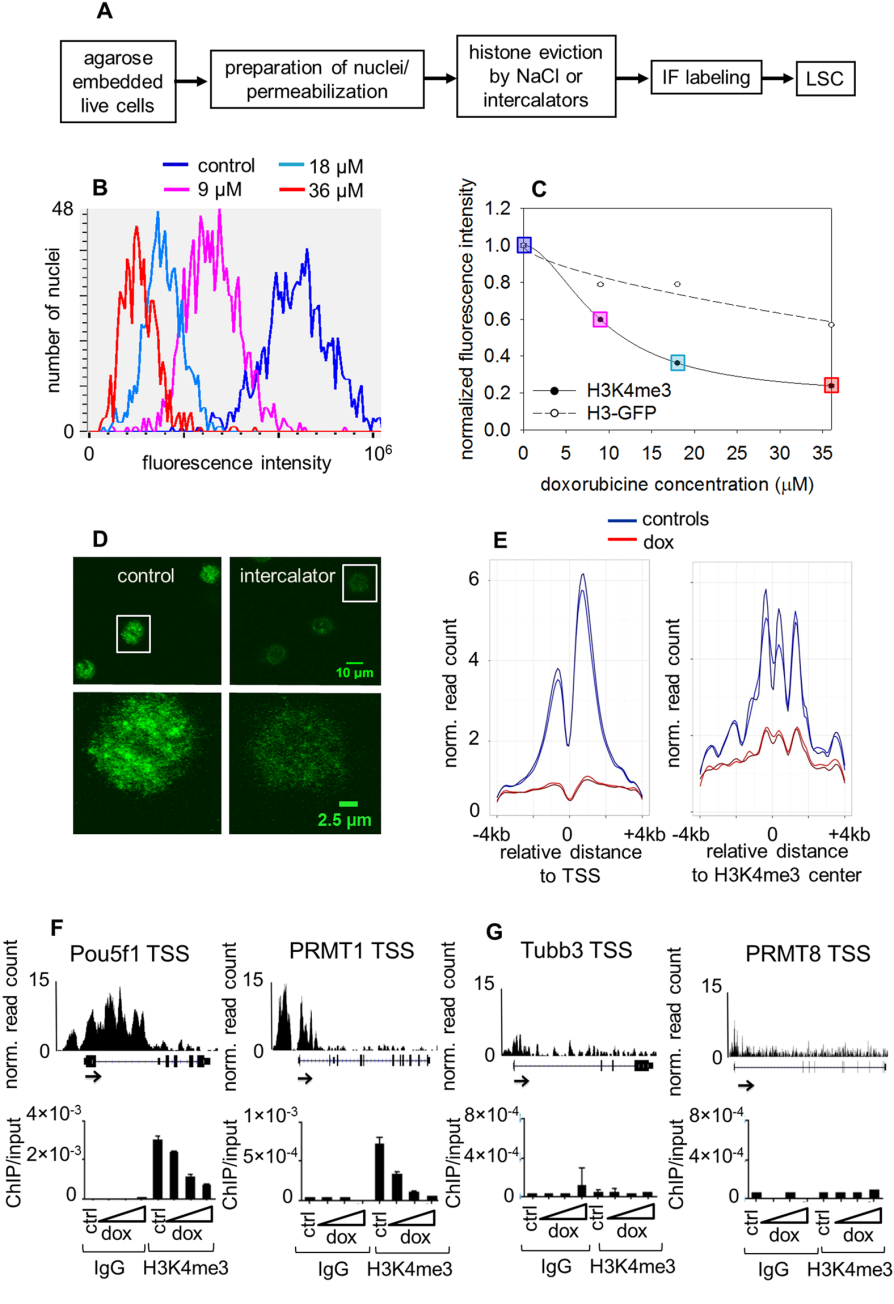
Doxorubicin induced eviction of nucleosomes. (**A**) Flow-chart of the method. Histones remaining in the nuclei after treatment with increasing concentration NaCl or intercalator solutions were detected by indirect immunofluorescence labeling and quantitatively analyzed by laser scanning cytometry (LSC). (**B**) Immunofluorescence intensity distribution histograms of H3K4me3 in the nuclei of control (blue) and doxorubicin treated G1 phase HeLa cells, using the following concentrations of the drug: 9 μM (magenta), 18 μM (light blue) and 36 μM (red). Integral fluorescence values for ~600 G1 nuclei were measured by LSC. Doxorubicin treatment was performed on live H3-GFP cells. The means of the H3-GFP signal (not shown in this panel) are plotted together with those of immunofluorescence on panel C. (**C**) Elution profiles constructed from the fluorescence distribution histograms generated by LSC. The curves demonstrate the decreasing levels of chromatin bound H3K4me3 (according to the color code used on panel B) and of the H3-GFP, used as internal reference, as a function of doxorubicin concentration. (**D**) H3K4me3 immunofluorescence staining of mES nuclei: representative CLSM images recorded at two magnifications, before (control) and after doxorubicin (intercalator) treatment. (**E**) ChIP-Seq density profiles of samples treated with 18 μM doxorubicin. H3K4me3 ChIP-seq analyses were performed in two technical replicates of the doxorubicine treated (red) and control (blue) samples. Anchor-plots of H3K4me3 sites around transcription start sites (TSSs; left) and around H3K4me3 positive non-TSS sites (right) are shown. ChIP-Seq signals were plotted in a ± 4 kb window. The Y axis shows the averaged read counts of the detected regions (tags normalized to 10 million). (See also: Supplementary Fig. S1A). (**F** and **G**, top) H3K4me3 genome browser images for two gene pairs expressed (F; Pou5f1 and PRMT1), and non-expressed (G; Tubb3 and PRMT8) in mES selected based on mES RNA-seq data (PRJNA302640). (**F** and **G**, bottom) ChIP-qPCR validation of the ChIP-Seq results shown in panel E at different doxorubicin concentrations (0 μM (ctrl), 6 μM, 9 μM and 18 μM; indicated by the gradient shape below the bar charts). Chromatin Immunoprecipitate (ChIP)/input ratios (referring to H3K4me3 vs mock IgG) are shown. (See also: Supplementary Fig. S1B–D).

We have developed a novel salt/intercalator elution based assay, Quantitative Imaging of the Nuclei after Elution with Salt/Intercalators (QINESIn) for the analysis of *in situ* nucleosome stability within intact nuclei. The workflow for the procedure (shown in Fig. 1A) involves: 1) elution of histones using either intercalators, or salt alone, to assess DNA superhelicity dependent and overall stability, respectively, and 2) measurement by LSC of the fraction of histones remaining chromatin-bound in the individual nuclei using histone type- or PTM-specific antibodies. Figure 1B demonstrates the fluorescence intensity distribution histograms of H3K4me3 carrying nucleosomes of nuclei treated with different intercalator concentrations recorded by LSC. Figure 1C shows the elution curve constructed from their means. When H3-GFP expressor cells are used, the GFP signal is measured in parallel with the immunofluorescence in each cell, serving as internal reference. Figure 1C demonstrates that the previously published observation that H3K4me3 carrying nucleosomes are more sensitive to eviction by doxorubicin intercalation in live cells than bulk H3 histones can be reproduced using our platform^43,58^. To avoid the redistribution of histones observed in those experiments^43^, permeabilized nuclei have been used in the experiments demonstrated below. The histones appear to be eluted in the nuclei in a homogeneous manner in the confocal microscope (Fig. 1D), but parallel ChIP-Seq measurements revealed that the H3K4me3 carrying promoter-proximal nucleosomes were more sensitive to doxorubicin treatment than those in regions outside TSSs (Fig. 1E, left and right panels; see also Supplementary Fig. S1A). These results were further validated using ChIP-qPCR conducted at different doxorubicin concentrations carried out on a pair of genes known to be expressed (Fig. 1F) and non-expressed (Fig. 1G) in mES cells, respectively. It was also demonstrated that doxorubicin itself was sufficiently washed out from the samples in the course of the experiment (Supplementary Fig. S1B) so as not to inhibit amplification and subsequent steps of the ChIP-Seq or ChIP-qPCR workflow (Supplementary Fig. S1C,D), a potentiality, stemming from an elevated melting temperature^55^, not considered and ruled out before^43^. The possibility of such interference was also excluded based on the fact that the amount of “input DNA” was not affected by doxorubicin treatment (data not shown). The above data confirm the relatively destabilized state of H3K4me3 modified nucleosomes using a novel approach, QINESIn, and reveal that the PTM itself may not be the sole factor in determining stability.

### Nucleosome stability measured by ethidium bromide elution

**Figure 2:**
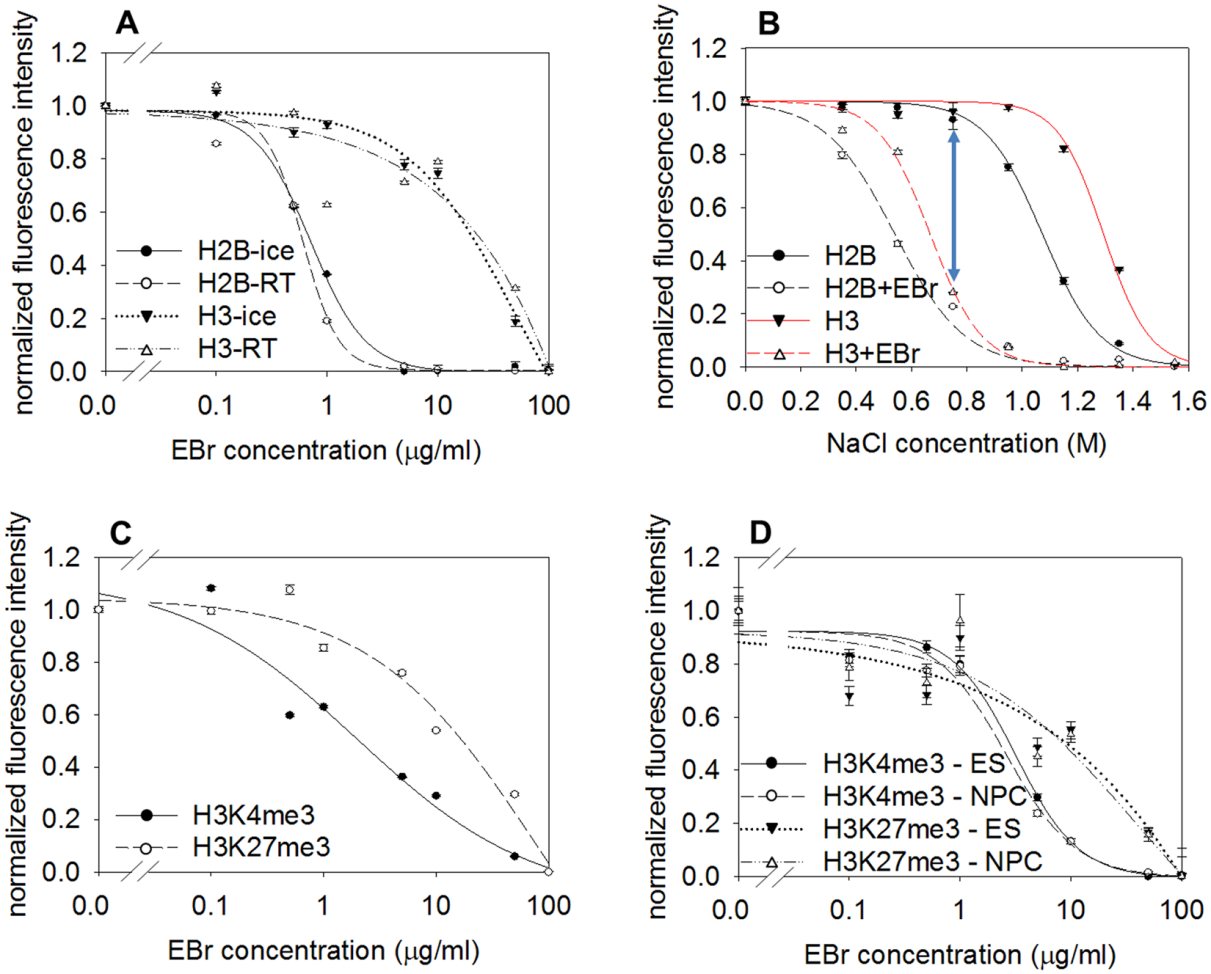
EBr induced nucleosome destabilization. (**A**) EBr induced elution of H2B-GFP and H3-GFP performed on ice and at room temperature (RT) in histone-GFP expressor HeLa nuclei. EBr was applied in the presence of 0.75 M NaCl. (**B**) Effect of co-treatment with EBr and salt in histone-GFP expressor HeLa nuclei. The shift of elution curves in the presence of 100 µg/ml EBr is seen comparing the continuous and dashed lines. The blue double arrow shows that the maximal effect of EBr on nucleosome stability occurs at 0.75 M salt. (**C**) EBr elution profiles of H3K4me3 or H3K27me3 in HeLa nuclei. (See also: Supplementary Fig. S1F). (**D**) EBr elution profiles of H3K4me3 or H3K27me3 in mES and NPC nuclei. (See also: Supplementary Fig. S2). The concentration of the intercalators are shown in a logarithmic scale in panels A, C and D. The elution curves refer to G1 phase cells gated according to their DNA fluorescence intensity distribution and the error bars represent SEM of ~600 G1 nuclei measured by LSC. The cell-to-cell and sample-to-sample C.V. values of the elution profiles are shown in Supplementary Table S1.

GFP-tagged versions of histones H2B and H3 exhibited differential doxorubicin sensitivity (Supplementary Fig. S1E), as expected^43^. However, the difference between the binding stability of the H2A-H2B dimer and that of the (H3-H4)_2_ tetramer was much more pronounced when another intercalator dye, EBr was used (Fig. 2A; note the log scale and the >20-fold difference between the concentrations of the intercalator required to elute 50% of H2B and H3). The elution profiles obtained with EBr were very similar at 4 °C and at RT. The dynamic range of EBr titration was best when it was added in the presence of 0.75 M salt where both histones are ~100% chromatin bound in the absence of EBr, while both come off almost completely in the presence of the high concentrations of EBr used (Fig. 2B). The differential eviction of H3K4me3 and H3K27me3-containing nucleosomes observed by doxorubicin intercalation in a more complex system^43^ could be reproduced using EBr and in circumstances when no reintegration of the histones was possible; the destabilized nature of H3K4me3 nucleosomes was demonstrated independently of cell type and differentiation state (in HeLa, in mES and in neural progenitor cells (NPC) differentiated from mES), as shown in Fig. 2C,D. Similar elution profiles were determined for H3K27me3 and H3-GFP, while H3K4me3 was significantly destabilized relative to H3-GFP, using H3-GFP as an internal control (compare Fig. 2C and Supplementary Fig. S1F). The patterns and extent of nuclear H3K4me3 staining were similar in all three cell types as shown in Supplementary Fig. S2A. The lack of a significant difference in the stability features of either H3K4me3 or H3K27me3 –containing nucleosomes between mES and NPC was unequivocally shown in experiments where mES and NPC samples, which had been differentially prelabeled in their plasma membrane with Alexa dyes, were mixed and analysed simultaneously in LSC (Fig. 2D; see experimental design in Supplementary Fig. S2B–E). The relatively destabilized character of the H3K4me3 nucleosomes was detected using two different monoclonal antibodies (Supplementary Fig. S3). The EBr elution profiles were remarkably reproducible (Supplementary Table S1).

To learn if the presence of reader proteins might affect the stability of H3K4me3 marked nucleosomes, measurements were conducted using a recombinant H3K4me3 reader protein, TAF3^61,62^. When the nuclei were pre-labeled with GST-tagged TAF3 and then exposed to intercalators (EBr, used at 750 mM salt; doxorubicin, used at 150 mM salt), those elution profiles were indistinguishable from what was measured using an anti-H3K4me3 antibody in the absence of the reader (Supplementary Fig. S4; both detected after elution by indirect immunoflurescence labeling). Thus, the stability features reflected by our assay appear to be nucleosome-autonomous.

The assay could also be performed on a flow-cytometric platform (Supplementary Fig. S5A) and in the confocal microscope, when the stability of H3K4me-marked nucleosomes at a specific chromatin region was compared to the stability of similar nucleosomes in the rest of the nucleus (Supplementary Fig. S5B). In the latter strategy an integrated lac operator (LacO) array was artificially trimethylated on the K4 lysine of H3 histones resulting in local chromatin decondensation^63^. The difference between the elution profiles measured over the whole nucleus and over the spot also suggests that the K4me3 PTM itself is not the sole determinant of nucleosome stability.

These data demonstrate the utility of EBr in intercalator elution to distinguish nucleosomes marked with different PTMs according to their stability features, extend the notion of H3K4me3-dependent relative instability to various cell types and differentiation states and impact on the understanding of the regulation involved.

### Nucleosome stability measured by salt elution

**Figure 3:**
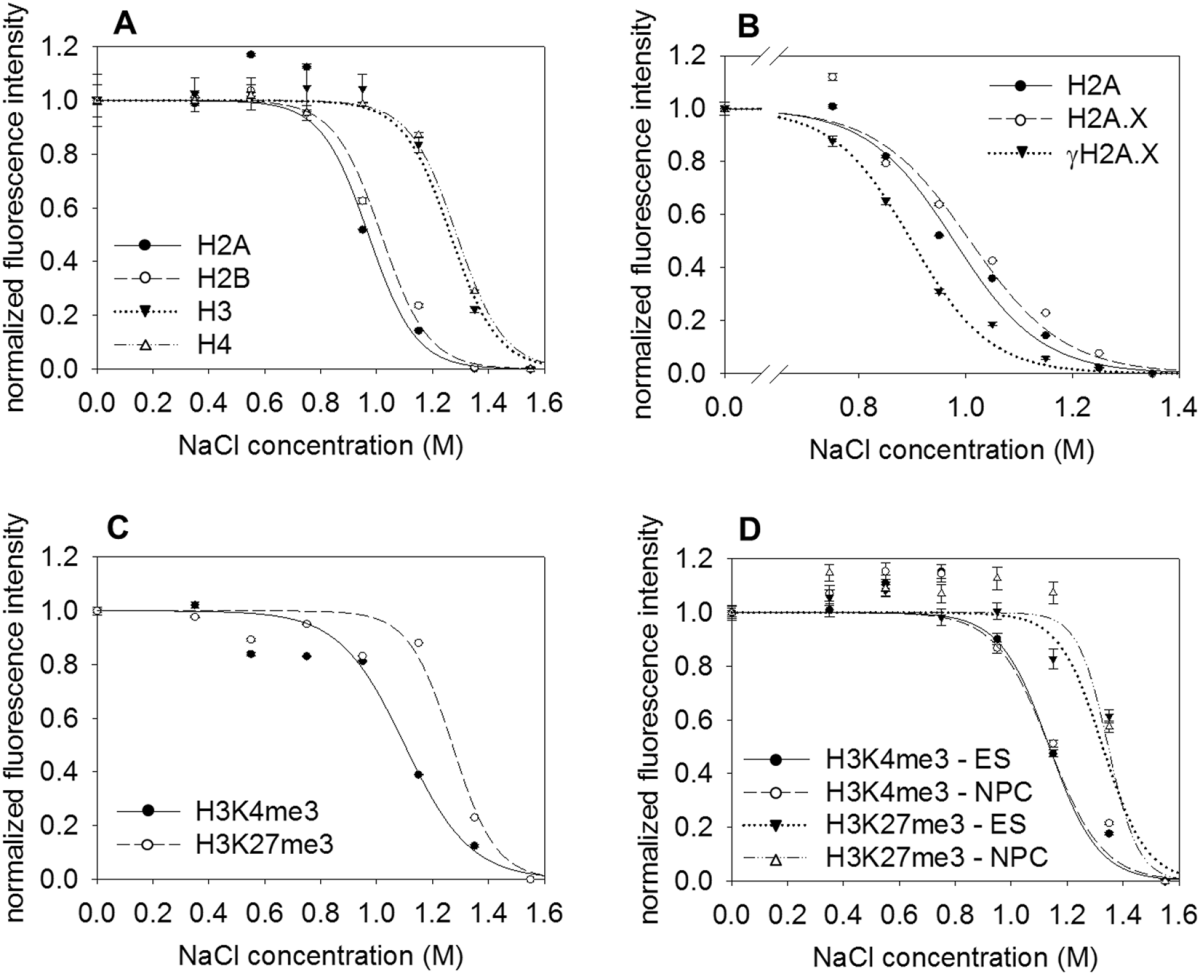
Salt induced nucleosome destabilization. (**A**) Salt elution profiles of antibody detected H2A, of H2B-GFP, H3-GFP and H4-GFP in histone-GFP expressor HeLa nuclei. (**B**) Salt elution profiles of γH2A.X, H2A.X and H2A, measured in parallel, in the nuclei of HCT116 cells exposed to 25 μM etoposide. (See also: Supplementary Fig. S6). (**C**) Salt elution profiles of H3K4me3 and H3K27me3 in HeLa nuclei. (See also: Supplementary Fig. S7). (**D**) Salt elution profiles of H3K4me3 and H3K27me3 in mES and NPC nuclei (See also: Supplementary Fig. S2). The elution curves refer to G1 phase cells gated according to their DNA fluorescence intensity distribution and the error bars represent SEM of ~600 G1 nuclei measured by LSC. The cell-to-cell and sample-to-sample C.V. values of the elution profiles are shown in Supplementary Table S1.

The well-known differential dissociation of the H2A-H2B dimer vs. the (H3-H4)_2_ tetramer from the nucleosomes by salt could also be monitored by the QINESIn platform: As shown in Fig. 3A, the assay was able to clearly distinguish between these two histone complexes in a salt elution format. The salt elution profiles were also well reproducible (see Supplementary Table S1), allowing detection of small differences, exemplified in the experiments shown in Fig. 3B. A significant destabilization of nucleosomes containing H2A.X was observed after phosphorylation of the histone variant upon brief exposure of cells to etoposide (focused on a narrower intercalator concentration range to increase precision). Etoposide, a topoisomerase II inhibitor, prevents the religation step of the enzymatic reaction leading to the accumulation of DNA double-strand breaks (see e.g.^64^) with consequential DNA damage response (DDR). For maximal accuracy, the experimental set-up was such that γH2A.X was measured simultaneously with H2A or H2A.X in the same sample. Etoposide increased the phosphorylated H2A.X levels above those of spontaneous γH2A.X (Supplementary Fig. S6A); the H2A.X vs. γH2A.X elution curves could be clearly distnguished at different time points of the early phase of DDR (Supplementary Fig. S6B–D). The histones were evicted from the nuclei in a homogeneous manner also upon elution by salt (Supplementary Fig. S6E).

The differences between the stability of H3K4me3 and H3K27me3 marked nucleosomes seen using intercalators were observed also via salt elution, in HeLa (Fig. 3C; for comparison of these data with the H3-GFP internal control see Supplementary Fig. S7), in mES as well as in NPC (Fig. 3D).

Thus, in its salt elution format, QINESIn was able to detect stability differences between H2A.X and γH2A.X containing nucleosomes and also to reveal the relativelíy destabilized nature of H3K4me3 in different cell types, showing that it is a rather general feature of these nucleosomes.

### The mechanisms of destabilization by intercalators and salt are intertwined

**Figure 4:**
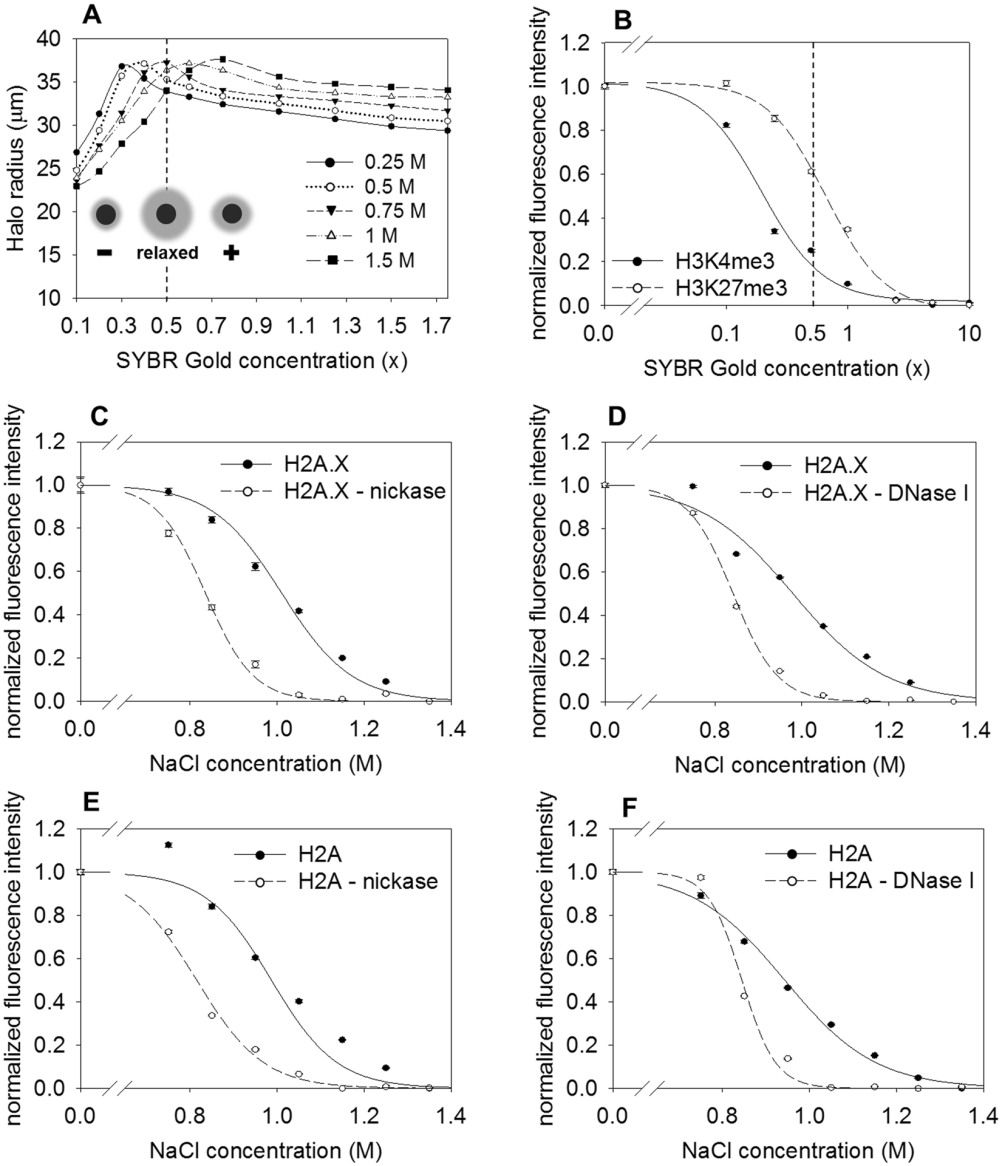
Superhelicity relaxation induced nucleosome destabilization. (**A** and **B**) Comparison of the changes of superhelicity (**A**) and nucleosome destabilization (**B**) in the case of the intercalator SYBR Gold, in Jurkat cells. (**A**) Determination of the relaxation concentration of SYBR Gold. The average halo radius of G1 phase cells was measured at increasing dye concentrations and in the presence of different salt concentrations (see “winding assay” in Methods). The inset shows the principle of the nuclear halo winding assay: As the intercalator concentration increases, the negatively supercoiled DNA loops get relaxed (halo size is increased), then overwound (i.e. becoming positively supercoiled with a decreased halo size). (**B**) Histone elution using SYBR Gold at 0.75 M NaCl. The SYBR Gold concentration inducing complete halo relaxation at 0.75 M NaCl concentration and where the DNA loops are completely relaxed is shown by the dashed line on both panels. (See also: Supplementary Fig. S8A) The concentration of the intercalators are shown in a logarithmic scale. Normalization was as described in the Methods. (**C** and **D**) Effect of nickase (**C**) and DNase I (**D**) treatment on H2A.X salt elution in HCT116 nuclei. (See also: Supplementary Fig. S8C and D). (**E** and **F**) Effect of nickase (**E**) and DNase I (**F**) treatment on H2A salt elution in HCT116 nuclei. (See also: Supplementary Fig. S8E and F). The elution curves refer to G1 phase cells gated according to their DNA fluorescence intensity distribution. Error bars represent SEM of ~600 G1 nuclei measured by LSC.

The strong salt dependence of the destabilizing effect of EBr suggested that the stability features dependent on DNA supercoiling and relying mainly on electrostatic interactions are interdependent. To reveal whether this effect is due to destabilization of electrostatic interactions involving the histones or the possible effect of salt on DNA relaxation per se, the effect of salt was measured on deproteinized nuclear halos in a sensitized supercoiling assay (Fig. 4A). SYBR Gold dye was applied as intercalator for this purpose, because its high quantum efficiency allowed accurate determination of the uphill part of the winding curves as well, which is not possible with EBr^65^. Using this dye and measuring the diameter of the nuclear halos by LSC, winding curves were recorded on the readily forming, large nuclear halos of Jurkat cells in the presence of different concentrations of NaCl. As shown in Fig. 4A, the ability of the intercalator to relax the DNA in the absence of histones was highly sensitive to salt concentration: The higher the salt concentration was, the more dye was necessary for relaxation. In contrast, intercalator induced nucleosome eviction was facilitated by salt, as shown in Fig. 2B. When SYBR Gold was applied in the presence of 0.75 M salt (included in the EBr elution experiments), the concentration of this intercalator that lead to near-complete eviction of H3K4me3 and ~50% eviction of H3K27me3 nucleosomes coincided with the SYBR Gold concentration at which maximal relaxation of the DNA loops occurred (Fig. 4B, Jurkat cells; Supplementary Fig. S8A, HeLa). Based on the above data we conclude that (1) nucleosomes are evicted by intercalators at superhelicity relaxation, (2) this is observed when the electrostatic interactions are also disturbed by salt. Figure 2B shows a similar collaborative effect: lower concentrations of salt are able to destabilize the nucleosomes in the presence of intercalators.

We argued that if intercalator induced nucleosome eviction is not just accompanied by but it is brought about by relaxation of superhelicity, then nicking the superhelical loops should destabilize nucleosomes. Indeed, when random single stranded breaks were introduced into the genomic DNA using a frequent cutter nickase enzyme, or by DNase I (Fig. 4C,E and D,F, respectively), the exogenous breaks significantly decreased nucleosomal binding of H2A and H2A.X during salt elution. Interestingly, this effect was not detected in intercalator elution (Supplementary Fig. S8B–F). Nickase and DNase I concentrations were used where the halo size was significantly increased without detectable loss of DNA content (Supplementary Fig. S9). In contrast with H2A and H2A.X, H3 exhibited higher sensitivity to nicking in the intercalator elution assay (Supplementary Fig. S10A,B).

Thus, there is a causal relationship between nucleosome destabilization and the superhelical relaxation induced by intercalators. The H2A-H2B dimers and the tetrasomes were differentially affected by relaxation.

## Discussion

The method described herein offers a simple means to assess and compare stability features of nucleosomes consisting of native histones *in situ*. Since the released nucleosomal components diffuse out of the measured volumes (as seen in Fig. 1D and Supplementary Fig. S6E), the relative sensitivity to salt or intercalators is assumed to be proportional to the off-rate term of the equilibrium dissociation constants involved in histone-histone and nucleosome-DNA interaction. Thus, an aspect of nucleosome stability which is pertinent to the principle of de-repression underlying gene regulation is addressed, in a PTM specific manner; PTMs are considered as important regulators of the DNA unwrapping dynamics^32^. The non-equilibrium conditions of the experimental setting upon elution discard the possibility of any repositioning of the released histones, which occurs upon supravital doxorubicin treatment^43,66^. Those chromatin regions where a particular PTM-marked histone has a higher-than-average off-rate (e.g. due to a second modification), will be eluted from a lower starting point; the contribution of the different regions to the elution profiles are not resolved in the assay performed by LSC.

The elution curves are independent of the expression levels of histones (Supplementary Fig. S11A,B) and are highly reproducible from experiment-to-experiment (see Supplementary Table S1). The permeabilized, hence ATP depleted, state of the nuclei is obviously incompatible with active chromatin remodelling processes, which is also evidenced from the lack of sensitivity of the method to temperatures between 0–22 °C during the elution and washing steps (Fig. 2A). The latter observation and the fact that the elution curves varied little when using 6 different permeabilizing buffers (Supplementary Fig. S11C) demonstrate the stability of the measuring conditions (see also sample-to-sample C.V. listed in Supplementary Table S1). As shown in Supplementary Table S1, the sample-to-sample variability of average nuclear immunofluorescence is 1.5–6x smaller than cell-to-cell variability. The latter values were very similar when whole IgG or its Fab fragments were used, suggesting that the width of the intensity distribution is unrelated to chromatin accessibility issues. In line with this conclusion, histone deacetylase inhibition didn’t increase labeling or change the C.V. significantly (data not shown). The width of the distribution of the ratio of two immunofluorescence signals, calculated separately for each cell (Supplementary Table S1), which is a parameter expected to be independent from potential cell-to-cell differences in antibody accessibility and binding, was very similar to those of the individual signals; this observation suggests that variability is more of biological than technical origin.

The suitable instrumental platforms of analysis include flow-cytometry (Supplementary Fig. S5A) and also confocal microscopy (Supplementary Fig. S5B). The iCys laser scanning cytometer might be replaced with other automated imaging platforms and software applications (see Materials and Methods, Automated microscopy). The fields of possible application can be further expanded by the fact that intercalator elution could also be performed on a flow-cytometric platform (Supplementary Fig. S5A), akin to the method that originally inspired the development of QINESIn^67^. However, such applications are limited to systems based on fluorescent protein tagged histones and could be used only at moderate salt concentrations that suspensions of nuclei can withstand.

The nucleosomal subpopulation measured by the QINESIn approach is, in the case of H3K4me3, rather homogenous. Since TSS proximal H3K4me3 comprises ~95% of all the H3K4me3 signals (based on our ChIP-Seq data, depending on the thresholds applied), the elution profile of H3K4me3 reflects the overall stability features of nucleosomes neighbouring active promoters, i.e. a rather well-defined subpopulation. In the case H3K27me3, overall stability features of a more heterogeneous population of nucleosomes belonging to facultative heterochromatin (see e.g.^68^) are revealed *en masse*, cell-by-cell. However, as Supplementary Fig. S5B shows, it is also possible to investigate localized chromatin domains *in situ*, what may include nuclear subregions or nuclear bodies.

The effects of intercalators like doxorubicin on chromatin structure^43,56–58^ are mainly attributed to their ability to modulate the twist of the mostly toroidal and constrained, negatively supercoiled nucleosomal DNA^53,69^, but the extent of modulation required for nucleosome destabilization and eviction has not been determined before. Using a sensitized winding assay we present experimental evidence that the superhelical DNA loops become maximally relaxed, but not yet overwound to form positive superhelices, at the concentration of intercalators where the bulk of nucleosomes are evicted (Fig. 4A,B). The destabilizing effects of nickase and DNase I treatments (Fig. 4C–F; Supplementary Fig. S10A) also support the conclusion that intercalators evict nucleosomes via the relaxation of superhelical twist and writhe.

The effect of EBr exhibits strong salt dependence (Fig. 2B), which may be the reason why eviction induced by EBr remained unrevealed in ref.^43^. In earlier works demonstrating EBr induced destabilization at low salt^19^, intercalator concentration exceeded that used by us. Additive destabilization of chromatin DNA-protein complexes by EBr and NaCl was also observed earlier^70^. The salt dependence of EBr effects could in part be explained by interactions mediated by the H1 histone expected to be completely released at 0.35 M salt (together with non-histone proteins^71,72^). It is feasible and necessary to further investigate this possibility using QINESIn.

DNA relaxation ensuing after either nickase or DNase I treatment results in a highly significant destabilization of the nucleosome, with different sensitivity to nicking in the salt and in the intercalator elution for H2A, H2A.X (Fig. 4C–F, Supplementary Fig. S8B–F), compared to H3 (Supplementary Fig. S10A,B). These observations emphasize that different determinants of nucleosome stability can be addressed by the two elution protocols, and the two formats of the assay may be differentially sensitive to different destabilizing effects. The QINESIn results are in line with biophysical data demonstrating that nucleosomes containing relaxed DNA exhibit enhanced sensitivity to salt^73^. Binding of an intercalator to the DNA relaxes and also extends superhelical DNA^43,53^; if the DNA is already relaxed (by nickase treatment), only extension is possible. Different contributions of these topological aspects to the binding strength of the different histones could explain the primarily salt- or intercalator-sensitivity of the response to nicking. The dramatic effect of nicking on nucleosomal stability confirms the direct coupling between DNA superhelicity and nucleosomal stability and suggests that modulation of superhelicity via transient nicking, by topoisomerases e.g., may be exploited by the cell for gene regulatory purposes, in line with models contemplated earlier^51^.

We have validated QINESIn with reference to published data^43^ and have confirmed them with the intercalator sensitivity of qPCR and ChIP-Seq being taken into account (see Supplementary Fig. S1B–D). The preferential sensitivity of H3K4me3 containing nucleosomes to doxorubicin eviction described in that publication (using MelJuSo cells and patients’ AML blasts) is corroborated here using HeLa, mES cells as well as NPC (Fig. 2C,D). These results indicate that the phenomenon is not unique to certain cell types and differentiation/proliferation states. The destabilized character of the H3K4me3 containing nucleosomes is in agreement with their high *in vivo* turnover^27^, suggesting that a physiologically relevant chromatin feature can be detected and analyzed by QINESIn. The fact that the PTM-specific differences manifested both in intercalator and salt elution experiments is in line with this interpretation.

The enhanced release of H3K4me3 upon intercalator treatment is apparently not due to the presence of a reader protein, what appears not to affect nucleosome stability based on the experiments with recombinant TAF3 (Supplementary Fig. S4), a reader protein that can be used to specifically label H3K4me3^61^. Its effect in TFIID recruitment can be reproduced by PHD domains of other high-affinity H3K4me3 binders^74^, so the lack of influence on the stability of the H3K4me3-marked nucleosomes may be a general feature of all the readers of this PTM. The modification itself is not expected to exert any direct effect on nucleosome structure and stability^48^; indeed, nucleosomes decorated with the same modification but outside TSSs were affected by doxorubicin in the same sample to a lesser degree (Fig. 1E right panel), suggesting that factors determined by the molecular environment of this PTM have a role in the striking H3K4me3 nucleosome destabilization. This conclusion is also supported by the fact that targeted H3K4 trimethylation in an artificial chromatin domain doesn’t lead to comparable destabilization (Supplementary Fig. S5B).

The PTM-specificity of the stability parameters detected with QINESIn is a powerful advantage of the approach. It will be of interest to extend the analysis to the characterization of nucleosomes containing other histone PTMs. It will be important to determine whether the destabilized nature of H3K4me3 marked, promoter proximal nucleosomes is related to the simultaneous presence of additional PTMs, e.g. acetylation, or to local topological factors.

This simple and versatile strategy is capable of resolving possible cell-cycle differences (Supplementary Fig. S12); however, such differences have not been observed in the case of the particular histones investigated in this study, suggesting that the degree of stability is a stable feature of the nucleosomes involved. The about twofold elevation of H3K4me3 levels in G2 cells (Supplementary Fig. S12, panel A) suggests that this PTM is copied over to the new nucleosomes upon DNA replication. A similar increment was found for another promoter proximal PTM recently^75^. In contrast, H3K27me3 shows no change in expression level across the cell cycle phases. The lack of any cell cycle dependence of the elution profiles implies that the stability of the new nucleosomes is similar or identical to the old ones.

The sensitivity of the method to structural changes of the histones is apparently determined by the effect of these changes on nucleosome stability; H2A.X, without phosphorylation, was indistinguishable from H2A in spite of the structural differences summarized in Supplementary Discussion. It is remarkable that γH2A.X, residing in repair foci (see Supplementary Fig. S6E), doesn’t become immobilized (Supplementary Fig. S6B–D) in these molecular aggregates. Apparently, the cohesion between the individual components of the foci is low enough to allow molecular exchanges, similarly to the lack of effect of H2A immune-cross-linking on H2B elution, and v.v. (Supplementary Fig. S13A,B). This is in line with the model that these foci are rather loose structures allowing access for the multitude of repair factors accumulating within the foci^76^. Moreover, γH2A.X is bound less tightly within nucleosomes than H2A.X, a difference detected only via salt elution (compare Fig. 3B and Supplementary Fig. S8B). Thus, QINESIn confirms the conclusion drawn from sedimentation velocity analyses^77^, indicating that phosphorylation on the C-terminus of H2A.X has a destabilizing effect on the nucleosome^78,79^, resolving a controversy^60^. Due to the fact that nucleosome stability is measured *in situ* rather than on reconstituted nucleosomes, the observed features may reflect the effect of several factors cooperating in determining nucleosome stability *in vivo*. Since the long stretches of chromatin packed with γH2A.X are generally visualized as being initiated at individual double-strand breaks^80^, the destabilized nature of γH2A.X nucleosomes may be due to these breaks, in line with the observations on the effect of nicking presented here. Although the degree of destabilization was smaller in the case of γH2AX as compared to the effect of nicking (compare Figs 3B and 4C), this may be due to heterogeneities within the γH2AX nucleosomes^81^, and/or the structural complexities of the repair foci. Destabilization may also be due to direct effects of the negative charge of the phosphate in γH2A.X, to the presence of protein factors bound on this platform^76^, to the PTMs associated with these variant histones (acetylation, ubiquitination)^22,40^, or any combination of the above. Destabilization of H2A.X nucleosomes after phosphorylation may be a medically relevant observation in the sense that DNA damage response could be further attenuated as a consequence of treatment with intercalating agents. Accurate comparison of the binding strength of H2A.X and γH2A.X was not possible in the context of the methodology applied in the study first reporting decreased DNA damage response in the wake of doxorubicin treatment^43^.

The main advantages of QINESIn over the other methods listed in Table 1 are the following. The conduct of nucleosomes containing fluorescent protein-tagged histones may be very different from features of the endogenous molecules: Supplementary Table S1 shows that the C.V. values calculated for the distribution of H2B-GFP or H3-GFP in the absence of any treatment tend to be larger than those obtained for the antibody detected histones. The effect of particular PTMs on nucleosome stability can be analyzed using reconstituted systems, if the enzyme complex generating that PTM is also available (see e.g. ref.^45^), but such measurements would address nucleosomes outside their chromatin context and would require the *in vitro* production of the modifying complex and a detailed understanding of its functioning. Our method is uniquely suitable for rapid screening of several features at the same time (e.g. to compare the stability of nucleosomes distinguished by different PTMs). Measurements can be readily conducted in microplates providing the method with high-throughput potential, sharply distinguishing QINESIn from the other approaches.

Native conditions may be approximated in the assay in view of the fact that the data presented here are in good agreement with the results obtained using different approaches in the literature and that different lysis conditions, including the one considered most physiological^82^, gave very similar elution profiles (Supplementary Fig. S11C). However, the relatively more unstable subpopulations of the nucleosomes studied may be underrepresented by the elution curves recorded in the case of unrecognized heterogeneities. This limitation can hardly be overcome in any experimental scenario addressing nucleosome stability (Table 1). When fixed cells (nuclei) are used (e.g. in ChIP-Seq-based approaches), i.e. the physiological conditions are supposed to be preserved, certain biochemical processes may occur before the fixed cellular structures are formed^83,84^ and fixation often affects antibody-antigen interactions what may give rise to differential representation of particular subpopulations. Another limitation of QINESIn when nucleosome stability is measured by salt or EBr is that several chromatin-associated proteins (including the linker histones) are released during the lysis/permeabilization process. The uncertainties related to this fact can be potentially overcome by the application of membrane permeable intercalators (like doxorubicin) that can be used in live cells; however, complexities arising from the possible reintegration of histones from the sites where they are evicted from should also be tackled.

Quantitative microscopy has proved useful in the epigenetics field as a tool of global analysis^85,86^; the method described here greatly extends its utility yielding data of direct functional relevance. Further examples of possible applications are described in the Supplementary Discussion.

## Methods

### Chemicals

All reagents were from Sigma-Aldrich (St. Louis, Missouri, USA) unless otherwise stated.

### Cell culture

HeLa cells expressing H2B-GFP, H3-GFP and H4-GFP fusion proteins^41^, LacO–I-SceI–TetO U2OS cells^63^ and HCT116 cells (Developmental Therapeutics Branch, National Cancer Institute, Bethesda, Bethesda, MD, 20892) were cultured in DMEM supplemented with 10% FCS, 2 mM l-glutamine, 100 μg/ml streptomycin, 100 U/ml penicillin. Jurkat cells were cultured in RPMI1640 medium supplemented with 10% FCS, 2 mM l-glutamine and antibiotics. Mouse embryonic stem cells (mES) were grown on 0.1% gelatin-coated plates in feeder-free condition. The mES medium was prepared by supplementing DMEM Glutamax medium with 15% FBS (Hyclone, South Logan, Utah, USA), 1000 U of leukemia inhibitory factor (LIF), 100 μg/ml streptomycin, 100 U/ml penicillin, non-essential amino acids and 2-mercaptoethanol.

### Neural differentiation

mES cells were differentiated into neural progenitor cells (NPC; compare Supplementary Fig. S2A middle and right panels) through embryoid body (EB) formation^87^. Briefly, for the induction of EB formation, cells were aggregated in 100 mm ⦰ bacteriological dishes (Greiner Bio-One International GmbH, Monroe, North Carolina, USA) at a density of 4 × 10^6^ cells/15 ml, in the absence of LIF. Over the span of four days, neural differentiation was induced by all-trans retinoic acid used at 5 µM final concentration. After four days of cultivation, trypsinized EBs were plated onto laminin/poly-L-ornithine precoated plates at a density of 1 × 10^5^ cells/cm^2^ in N2 medium^87^.

### Embedding live cells into low melting point agarose

Prior to embedding, the wells of 8-well chambers (Ibidi, Martinsried, Germany) were coated with 1% (m/v) low melting point (LMP) agarose. 150 μl liquid agarose, diluted in distilled water, was dispensed into each well and was immediately removed so that a thin agarose layer remained on the surfaces and was left to polymerize on ice for 2 minutes, then kept at 37 °C until the surface of the wells dried out. This coating procedure was repeated once more on the same chambers. Embedding was performed keeping cells and agarose at 37 °C. The cell suspension containing 6 × 10^6^ cells/ml was mixed with 1% LMP agarose diluted in 1 × PBS (150 mM NaCl, 3.3 mM KCl, 8.6 mM Na_2_HPO_4_, 1.69 mM KH_2_PO_4_, pH 7.4) at a v/v ratio of 1:3. 22 μl of the cell-agarose suspension was dispensed in the middle of the wells and the chambers were covered with home-made rectangular plastic coverslips (Supplementary Fig. S13C) cut out from a medium weight polyvinyl chloride binding cover of 200 μm thickness (Fellowes, Inc., Itasca, Illinois, USA). The cells were left to sediment on the surface of the coated wells for 4 minutes at 37 °C, then kept on ice for 2 minutes. After polymerization of the agarose, 300 μl ice cold complete culture medium was added to each well, a step aiding removal of the coverslips.

### Preparation of nuclei/permeabilization and histone eviction by salt or intercalators

The agarose-embedded cells at the bottom of the wells were washed with 500 μl ice cold 1 × PBS, three times for three minutes, then permeabilized with 500 μl ice cold 1% (v/v) Triton X-100 dissolved in 1 × PBS/EDTA (5 mM EDTA in PBS), for 10 minutes. This step was repeated once more. After permeabilization, nuclei were washed with 500 μl ice cold 1 × PBS/EDTA three times for three minutes and were treated with different concentrations of NaCl or intercalator solutions on ice. Ethidium bromide (EBr) and SYBR Gold (supplied by the manufacturer, Thermo Fisher Scientific (Waltham, Massachusetts, USA), as 10,000 x stock solution) were diluted in 1 × PBS/EDTA supplemented with 600 mM NaCl, to the final concentrations indicated in the Figures. EBr was used at 100 μg/ml when the salt concentration was titrated (Fig. 2B). Doxorubicin (TEVA, Debrecen, Hungary) was diluted and added to live cells in complete DMEM medium, or diluted in 1 × PBS/EDTA when added to the permeabilized cells. Nuclei were washed with 400 μl of ice cold salt or intercalator solution six times, for 10 minutes. After this step, the nuclei were washed with 500 μl ice cold 1 × PBS/EDTA three times for 3 minutes. Since NaCl was diluted in 1 × PBS/EDTA, the salt concentrations indicated on the X axes of the graphs in all the Figures show the total NaCl concentrations together with NaCl present in the PBS buffer. Analysis of the curves was made by SigmaPlot 12.0, using either ‘Sigmoid 3 parameter’ (in the case of linear plots) or ‘Standard curves: Four Parameter Logistic Curve’ (in the case of logarithmic plots) curve-fitting subroutines. Fitting was not applied to the data points in Fig. 4A. Elution curves were normalized to ‘0’ substracting the smallest value from all the others, and to ‘1’ dividing the mean fluorescence intensities represented by the data points by that of the non-treated sample. At the experiments where eviction was partial, normalization was performed only for ‘1’. The number of analyzed G1 nuclei were between 200–1000/well, out of the about 500–2000 cells scanned. All the SEM values indicated in the Figure legends were calculated from the datapoints of the cell population analyzed in the given experiment.

### Immunofluorescence labeling

After salt or intercalator treatment, the samples were incubated with 500 μl 5% (m/v) Blotto Non-Fat Dry Milk (Santa Cruz Biotechnology Inc., Santa Cruz, California, USA) in 1 × PBS/EDTA for 30 minutes on ice, to decrease nonspecific binding of the antibodies. The blocking solution was washed out with 500 μl ice cold 1 × PBS/EDTA three times for three minutes and indirect immunofluorescence labeling was performed using rabbit polyclonal anti-H2A (Abcam, Cambridge, UK; 0.4 mg/ml), rabbit polyclonal anti-H2A.X (Abcam, Cambridge, UK; 1 mg/ml), mouse monoclonal anti-γH2A.X (Merck-Millipore, Darmstadt, Germany; 1 mg/ml), mouse monoclonal anti-H3K4me3 (ref.^38^; 0,5 mg/ml) or mouse monoclonal anti-H3K27me3 (ref.^88^; 0,5 mg/ml) primary antibodies, all diluted in 150 μl of 1 × PBS/EDTA/1% BSA (1 × PBS/EDTA supplemented with 1% w/v bovine serum albumin), at 4 °C, overnight. All the above antibodies were applied to the wells at a titer of 1:800. After labeling with the primary antibodies, the nuclei were washed with 500 μl ice cold 1 × PBS/EDTA three times for 10 minutes. Labeling with the secondary antibodies was performed in 150 μl 1 × PBS/EDTA for two hours on ice, using Alexa fluor 488 (A488) or Alexa fluor 647 (A647) conjugated goat anti-mouse IgG or goat anti-rabbit IgG antibodies (Thermo Fisher Scientific, Waltham, Massachusetts, USA). The secondary antibodies were also used at a titer of 1:800, diluted in 1 × PBS/EDTA from 2 mg/ml stock solutions. After labeling with the secondary antibodies, the agarose-embedded nuclei were washed with 500 μl ice cold 1 × PBS/EDTA three times for 10 minutes. Then the samples were fixed in 1% formaldehyde (dissolved in 1 × PBS/EDTA) at 4 °C, overnight. After fixation, the wells containing the embedded nuclei were washed with 500 μl ice cold 1 × PBS/EDTA three times for 3 minutes and were stained with 200 μl 12.5 μg/ml propidium–iodide (PI, dissolved in 1 × PBS/EDTA) for 30 minutes, on ice. The stained nuclei were washed three times with 500 μl ice cold 1 × PBS/EDTA for 3 minutes. Fluorescence intensity distributions were recorded using an iCys laser scanning cytometer (LSC), as described below. When histone-GFP expressor cells were used, the GFP signal served as internal reference (characterizing the overall features of H3 or H2B) for the PTM-specific immunofluorescence measured simultaneously in each cell.

### Comparison of mouse embryonic stem cells and neuronal progenitor cells in a mixed sample

Experimental conditions were elaborated for an accurate comparison of nucleosome stability in a mixture of cells (data in Figs 2D, 3D, Supplementary Fig. S2). Before embedding into agarose, cell membrane proteins of live ES and NPC cells were labeled nonspecifically with two different Alexa fluorophores (Alexa fluor 488; 5,5 mg/ml in DMSO and Alexa fluor 647; 10 mg/ml in DMSO; Thermo Fisher Scientific, Waltham, Massachusetts, USA). 1.5 × 10^6^ live ES and NPC cells were incubated with Alexa fluor 488 or Alexa fluor 647, respectively, in 1 ml 1 × PBS at a titer of 1:2500, under constant shaking at 37 °C, for 30 minutes. Labeled cells were washed three times with 5 ml 1 × PBS, mixed 1:1, embedded into agarose and the fluorescence signals of the two dyes were recorded by LSC to identify individual ES and NPC cells in the wells. Then, the membraneous structures of the cells (carrying most of the Alexa labelling) were completely (see Supplementary Fig. S2B) removed by detergent treatment (i.e. permeabilization with Triton X-100, as described above). After salt or EBr elution, indirect immunofluorescence labeling of H3K4me3 (using mouse monoclonal anti-H3K4me3; Abcam, Cambridge, UK) and of H3K27me3 (using rabbit monoclonal anti-H3K27me3; Cell Signaling Technology Inc.; Danvers, Massachusetts, USA) histones was performed, using the secondary antibodies at a titer of 1:800. In a second LSC scan, labeled histones were detected and, using a merging process, fluorescence intensities of the histones were assigned to ES and NPC cells identified previously in the first run.

### Etoposide treatment

Agarose embedded live cells were treated with etoposide (TEVA, Debrecen, Hungary), used at a final concentration of 25 µM. The drug was diluted in 300 µl complete DMEM medium and the cells were incubated together with the drug at 37 °C in 5% CO_2_ atmosphere, for 1, 3 or 6 hrs.

### Nickase and DNase I treatment

Live cells were embedded into agarose as described above and treated with 500 μl ice cold lysis buffer (0.4% (v/v) Triton X-100, 300 mM NaCl, 1 mM EDTA, 10 mM Tris-HCl, pH8) for 10 minutes, followed by treatment with 500 μl ice cold 1% (v/v) Triton X-100 dissolved in 1 × PBS/EDTA, for 10 minutes, then washed three times with 500 μl ice cold 1 × PBS/EDTA. The frequent cutter Nt.CviPII nickase (recognition site: CCD; New England Biolabs Inc., Ipswich, Massachusetts, USA) and DNase I were applied after the washing steps following permeabilization (see above). Before digestion, the samples were equilibrated with nickase buffer (10 mM Tris-HCl pH 8, 50 mM NaCl, 10 mM MgCl_2,_ 1 mg/ml BSA) or with DNase I buffer (10 mM Tris-HCl pH 8, 0.1 mM CaCl_2_, 2.5 mM MgCl_2_) by washing three times with 500 µl of the buffer solutions. Nickase treatment was performed in 300 µl nickase buffer for 30 min at 37 °C, using the enzyme at a final concentration of 0.5 U/ml, titrated so as to maximize nuclear halo radii without losing DNA (as shown in Supplementary Fig. S9A and B). DNase I digestion was performed in 300 µl DNase I buffer for 10 min at 37 °C, at a final concentration of 0.1 μg/ml DNase I; in these conditions both single and double stranded breaks can be expected, but no significant loss of nuclear DNA content was seen (Supplementary Fig. S9C). After enzymatic treatments, the samples were washed with 500 μl ice cold 1 × PBS/EDTA three times for three minutes.

### Determination of the relaxation concentration of intercalators

Jurkat cells (1000 cells/μl) were lysed in an isotonic lysis buffer (Tris-HCl 20 mM, pH 7.0, sucrose 300 mM, EDTA 5 mM, EGTA 1 mM, spermine 1 mM, Triton X-100 0.2% (v/v)) on ice for 7 minutes. The same buffer without Triton X-100 was added to the lysate to achieve a 10 nuclei/μl final concentration and the lysate was transferred in 100 μl aliquots into the wells of an α-poly-L-lysine (MW 150 000–300 000) coated 96 well plate (TPP, Switzerland), then spun down (750 g, 10 minutes, 4 °C). From each well, 70 µl aliquots were removed and 270 μl ice cold nuclear extraction buffer (Tris-HCl 20 mM, pH 7.5, NaCl 2.22 M, EDTA 5 mM, EGTA 1 mM) was added to remove the histones. Then 270 μl portions were carefully removed from the supernatant and 270 μl winding solution (Tris-HCl 20 mM, pH 7.5, EDTA 5 mM, EGTA 1 mM) supplemented with NaCl and SYBR Gold so as to produce the final concentrations represented on Fig. 4A was added. The resulting nuclear halos were then analyzed using LSC. The procedure is mentioned briefly as “winding assay” in the Figure 4A legend.

### Automated microscopy

Automated microscopic imaging was performed using an iCys instrument (iCys® Research Imaging Cytometer; CompuCyte, Westwood, Massachusetts, USA). Green fluorescent protein (GFP), SYBR Gold, A488, doxorubicin and PI were excited using a 488 nm Argon ion laser, A647 was excited with a 633 nm HeNe laser. The fluorescence signals were collected via an UPlan FI 20 × (NA 0.5) objective. GFP and A488 were detected through 510/21 nm and 530/30 nm filters, respectively, while doxorubicin, A647 and PI were detected through a 650/LP nm filter. Each field (comprising 1000 × 768 pixels) was scanned with a step size of 1.5 µm. In the case of the winding assay performed on nuclear halos, SYBR Gold fluorescence was collected via a 10x objective and detected through a 550/30 nm filter. Amplification of the SYBR Gold signal via PMT voltage and gain were adjusted for each well separately so that the matrix area of the G1 phase halos (at a threshold value of 4000 pixel intensity) was kept constant. Background subtraction (offset) was adjusted so that the background pixel intensity was set around 300 in each well. The average halo radii for G1 cells were calculated from the area of the halos (measured at a threshold value of 600 pixel intensity). Data evaluation and hardware control were performed with the iCys 7.0 software for Windows XP. Gating of G1 phase cells was according to the fluorescence intensity distribution of the DNA labeled with PI (see Supplementary Fig. S2D,E) or SYBR Gold (not shown). (The iCys laser scanning cytometer can be replaced with other automated imaging platforms and softaware applications (e.g. Olympus ScanR, Molecular Device ImageXpress, Perkin Elmer Opera Phenix, ThermoFisher CellInsight, GE IN Cell Analyzer). When appropriate image series are generated, software applications (like CellProfiler, Fiji, ImageJ, Micropilot) are readily available for their high content analyses.)

### Chromatin immunoprecipitation and sequencing

Chromatin immunoprecipitation and sequencing (ChIP-Seq) and chromatin immunoprecipitation-quantitative PCR (ChIP-qPCR) experiments were carried out as in^89^, with minor modifications. Briefly, nuclei were cross-linked in 4% methanol-free ultrapure formaldehyde (Thermo Fisher Scientific, Waltham, Massachusetts, USA) for 10 minutes at room temperature. Glycine was added for 5 min at a final concentration of 125 mM. After fixation, chromatin was sonicated with a Diagenode Bioruptor to generate 200–1000 bp fragments. Chromatin was immunoprecipitated with rabbit polyclonal anti-H3K4me3 (Abcam, Cambridge, UK; 1 mg/ml) antibody using pre-blocked magnetic beads (Dynabeads Protein A, Thermo Fischer). Eluted DNA was purified (MinElute PCR Purification Kit; Qiagen Inc., Valencia, California, USA), then quantified with a Qubit fluorometer (Thermo Fisher Scientific, Waltham, Massachusetts, USA). ChIP-seq libraries were prepared from two biological replicates by Illumina according to manufacturer’s instructions.

### Bioinformatics analysis

Primary analysis of the ChIP-Seq raw reads was carried out using a ChIP-Seq analyze command line pipeline^90^. Briefly, Burrows-Wheeler Alignment Tool (BWA)^91^ was used to align the reads to the hg19 genome assembly (GRCh37), with default parameters. Histone regions were detected by findPeaks.pl (with options ‘-region’, ‘-style histone’, ‘-size 1000’ and ‘-minDist 2500’). Intersections, subtractions and merging of the predicted peaks were made using BedTools. The center of the ChIP-Seq distributions of the TSS-negative H3K4me3 regions was found using getPeaks from Homer with option’-nfr’. Control and doxorubicin treated H3K4me3 samples were analyzed by DiffBind v1.0.9 (with parameters ‘minOverlap = 2’ and ‘full library size’^92^), using duplicates. Genome coverage files (bedgraph files) for visualization were generated by makeUCSCfile.pl and then converted into tdf files using igvtools with ‘toTDF’ option. Integrative Genomics Viewer (IGV 2.3, Broad Institute) was used for data browsing^93^. Normalized tag counts for Meta histogram and Read Distribution (RD) plots were generated by annotatePeaks.pl with options ‘-ghist’ and ‘-hist 25’ from HOMER and then visualized by R using or Java TreeViewer.

## Data Availability Statement

The datasets generated during and/or analysed during the current study are available from the corresponding author on reasonable request. ChIP-Seq data are available on BioProject databank (Accession: PRJNA360561)

## Electronic supplementary material

Supplementary Materials
