# Supplementary material for: Nucleosome stability measured in situ by automated quantitative imaging
Source: Sci Rep. 2017 Oct 6;7:12734. doi: 10.1038/s41598-017-12608-9 (PMC5630628; doi:10.1038/s41598-017-12608-9)
Supplement: Supplementary file 1 — Supplementary Materials [file 41598_2017_12608_MOESM1_ESM.doc]

**Nucleosome stability measured in situ by automated quantitative imaging**

László Imre^1^, Zoltán Simándi^2,6^, Attila Horváth^2^, György Fenyőfalvi^1^, Péter Nánási^1^, Erfaneh Firouzi Niaki^1^, Éva Hegedüs^1^, Zsolt Bacsó^1^, Urbain Weyemi^3^, Rebekka Mauser^5^, Juan Ausio^7^, Albert Jeltsch^5^, William Bonner^3^, László Nagy^2,6,8^, Hiroshi Kimura^4^ and Gábor Szabó^1^,*

^1^ Department of Biophysics and Cell Biology, University of Debrecen, Debrecen, H-4032, Hungary

^2^ Department of Biochemistry and Molecular Biology, University of Debrecen, Debrecen, H-4032, Hungary

^3^ Center for Cancer Research National Cancer Institute, Bethesda, Maryland, 20892, USA

^4^ Cell Biology Unit, Institute of Innovative Research, Tokyo Institute of Technology, Yokohama, 226-8501, Japan

^5^ Institute of Biochemistry, Stuttgart University, Stuttgart, Germany

^6^ Sanford Burnham Prebys Medical Discovery Institute, Orlando, Florida, USA

^7^ University of Victoria, Department of Biochemistry, Victoria, BC, V8W 3P6, Canada

^8^MTA-DE “Lendulet” Immunogenomics Research Group, University of Debrecen, Debrecen, Hungary

*Corresponding author: Gábor Szabó. Tel: +36-52-412-623/65119; Fax: +36 52 532 201/66726; email: szabog@med.unideb.hu

**SUPPLEMENTARY FIGURES AND TABLES**


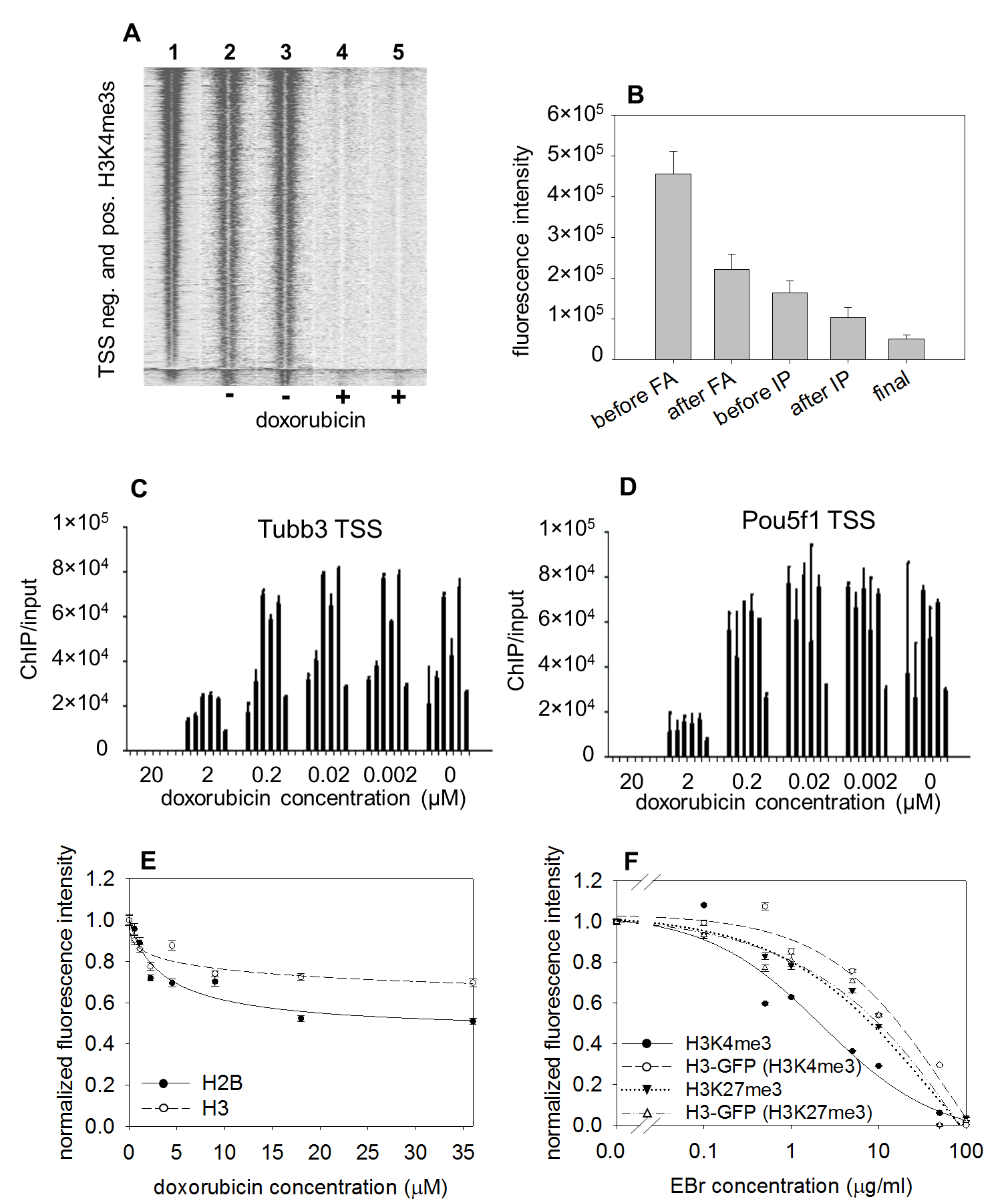


Supplementary Fig. S1

Additional data and control measurements to the experiment in Fig. 1E-G and the internal controls of the H3K4me3/H3K27me3 EBr elution measurements in Fig. 2C.

(A) ChIP-Seq read distributions of the experiment in Fig. 1E. Lanes 2 and 3: technical replicates of control samples; lanes 4 and 5: replicates of doxorubicin treated samples of the current experiment. Control H3K4me3 signals from an independent experiment are shown in lane 1.

(B) The release upon ChIP washing steps (described in ref.[^1^](#_ENREF_1)) of intercalated doxorubicin measured in agarose embedded HeLa nuclei by LSC. Doxorubicin fluorescence was followed through the consecutive steps of the ChIP protocol: before and after formaldehyde fixation (FA), before and after immunoprecipitation (IP) and at the end of the protocol („final”). SDS was excluded from the buffers because the ionic detergent completely desintegrated the nuclei. Error bars represent SEM of ~600 G1 nuclei measured by LSC. (See also: Fig. 1E)

(C and D) Effect of doxorubicin on the efficiency of qPCR reactions. Doxorubicin was added to

the input DNA at the final concentrations indicated and incubated for 15 minutes at RT prior to addition of an equal volume of the qPCR mastermix. Error bars represent the SD of 6 parallel samples. (See also: Fig. 1F, G)

(E) Doxorubicin induced H2B-GFP and H3-GFP elution in histone-GFP expressor HeLa nuclei.

(F) H3-GFP elution curves serving as internal control in the experiment of Fig. 2C. H3-GFP data points are plotted together with the curves of Fig. 2C. The concentration of the intercalators are shown in a logarithmic scale.

The curves on panels E and F refer to G1 phase cells gated according to their DNA fluorescence distribution. Error bars represent SEM of ~600 G1 nuclei measured by LSC.


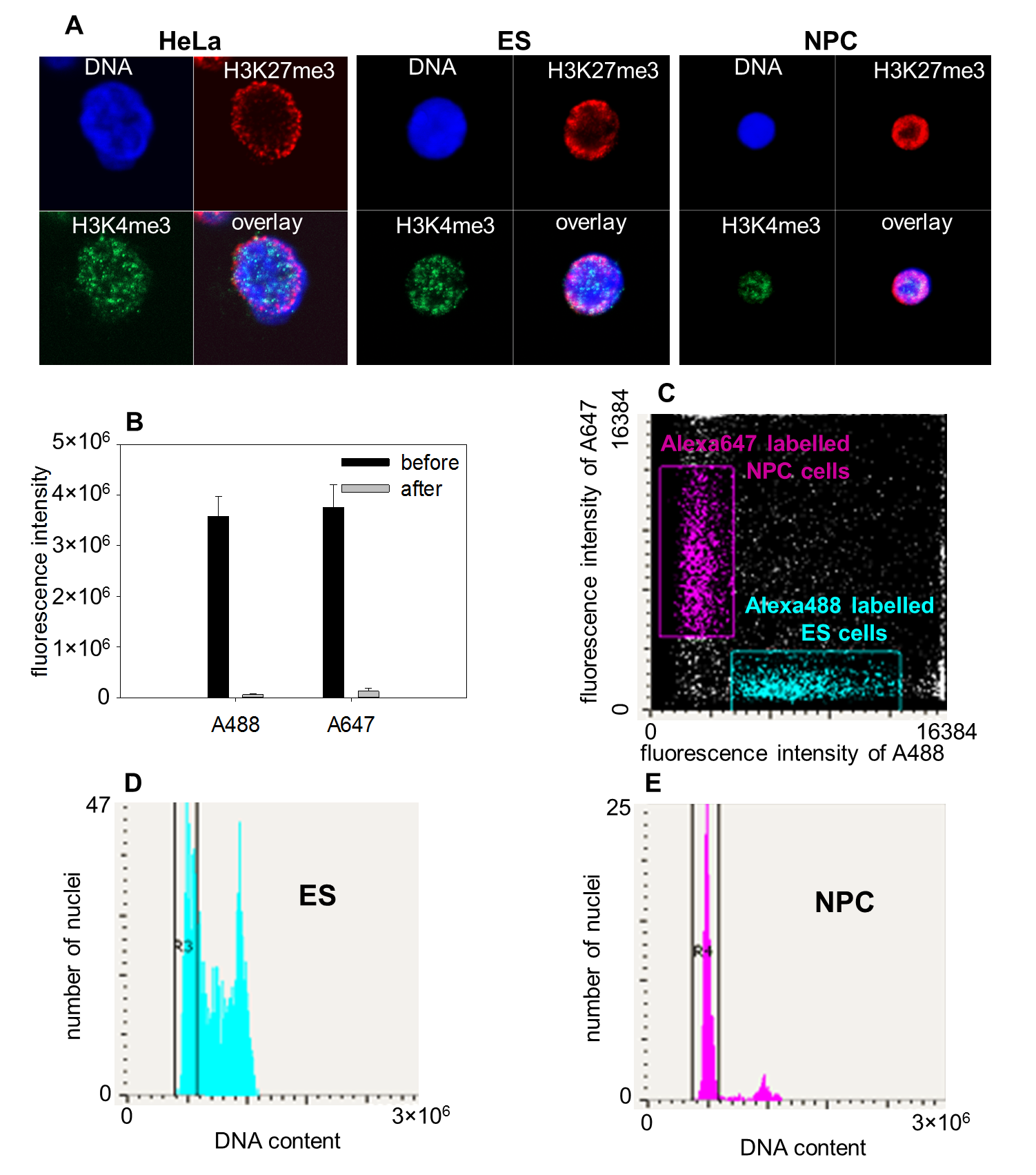


Supplementary Fig. S2

Control studies in support of the mixed cell experiment shown in Figure 2D and Figure 3D.

(A) Nuclear localization of H3K4me3 and H3K27me3 fluorescence signals in HeLa, ES and NPC nuclei (CLSM images).

(B) Model experiment using Jurkat cells demonstrating the lack of interference of cell membrane prelabeling with the subsequent, post-permeabilization visualization of histones. The mean fluorescence intensity of cells prelabeled live with two Alexa dyes (A488 or A647) was recorded before (black columns) and after (grey columns) cell lysis with Triton X-100.

(C-E) Gating protocol used in the experiments of Fig. 2D and Fig. 3D.

(C) The mixed cell population of A488 prelabeled mES and A647 prelabeled NPC cells were gated so as to be distinguished in the subsequent elution experiment.

(D and E) G1 phase nuclei of mES and NPC cells, distinguished from each-other by their A488 or A647 fluorescence (before the addition of TX100, as shown in (C)), were further gated (after TX100) based on the DNA distribution histograms.


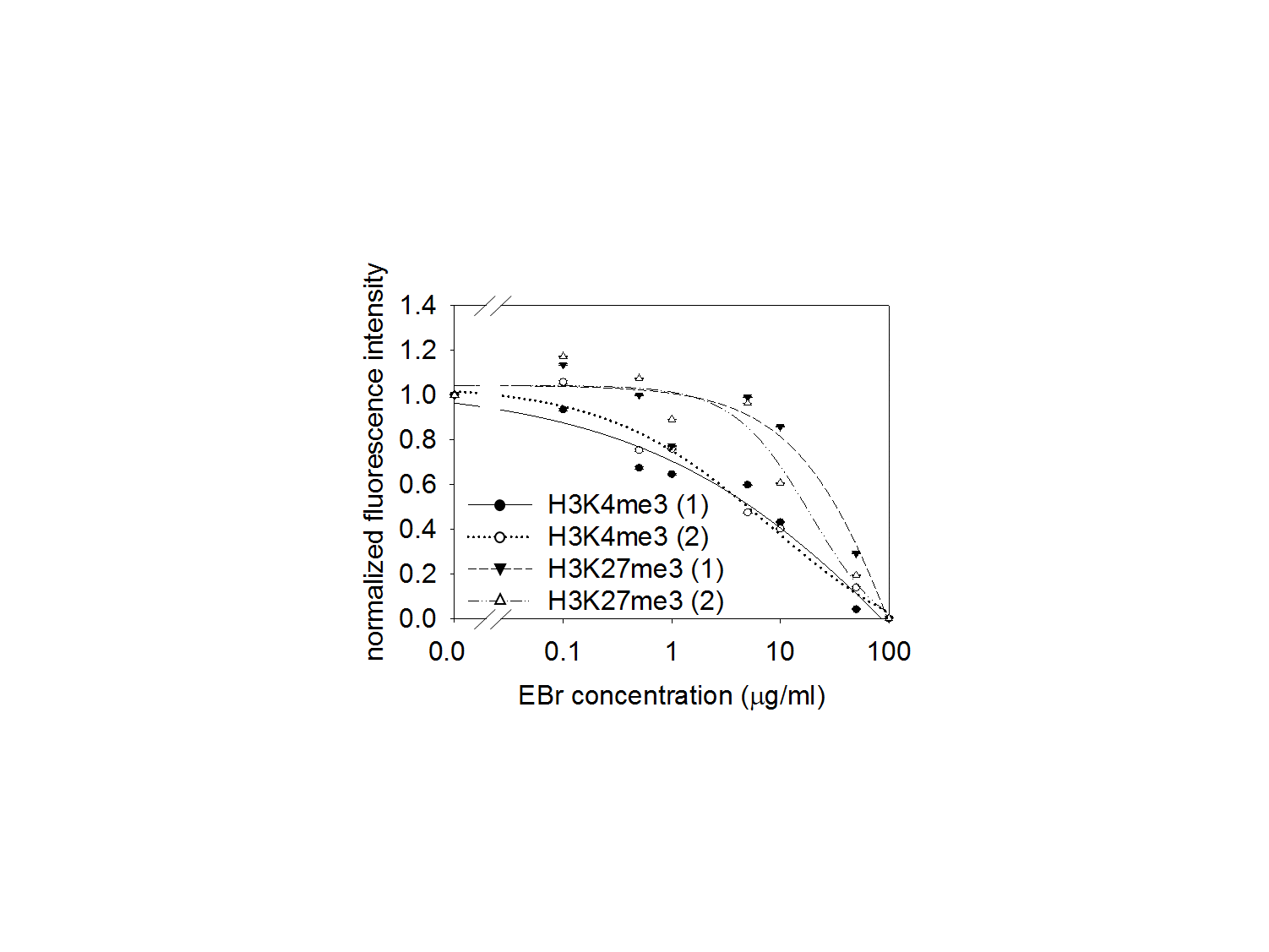


Supplementary Fig. S3

Comparison of EBr elution of H3K4me3 and H3K27me3 measured in HeLa nuclei, using antibodies from different manufacturers.

H3K4me3: (1) mouse monoclonal anti-H3K4me3 (Abcam); (2): mouse monoclonal anti-H3K4me3 (ref.[^2^](#_ENREF_2)); H3K27me3: (1) rabbit monoclonal anti-H3K27me3 (Cell Signaling Technology); (2) mouse monoclonal anti-H3K27me3 (ref.[^3^](#_ENREF_3)).

The concentration of the intercalators are shown in a logarithmic scale. Error bars represent SEM of ~600 G1 nuclei measured by LSC.


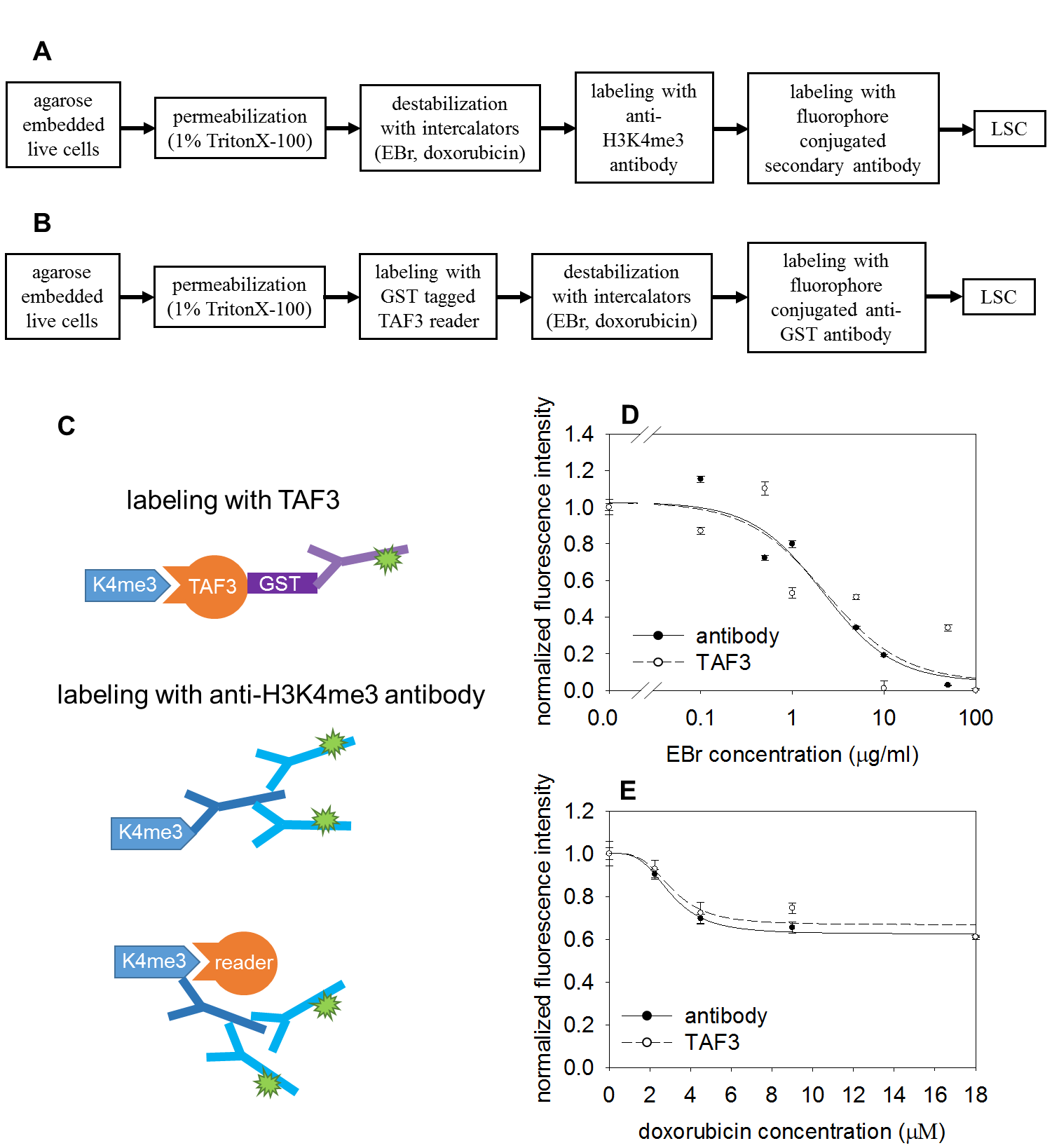


Supplementary Fig. S4

H3K4me3 and TAF3 intercalator elution experiments in HeLa nuclei.

(A and B) Flow-chart of the H3K4me3 (A) and TAF3 (B) intercalator elution experiment

(C) Schemes of H3K4me3 labeling using the GST-tagged PHD domain of the reader protein TAF3, and by an anti-H3K4me3 antibody.

(D) H3K4me3 intercalator elution curves obtained using either anti-H3K4me3 primary antibody (ref.[^2^](#_ENREF_2)) or a GST-tagged TAF3. The latter was added to the nuclei pior to intercalator treatment (as indicated in the flow-chart on panel B). Following elution, the levels of chromatin-bound H3K4me3 were detected in the two samples („antibody” and „TAF3”) using dye-conjugated goat anti-mouse secondary or anti-GST antibody, respectively. The concentration of the intercalators are shown in a logarithmic scale.

(E) The same experiment as in (D) but with doxorubicin used as the intercalator.

The curves refer to G1 phase cells gated according to their DNA fluorescence distribution. Error bars represent SEM of ~600 G1 nuclei measured by LSC.


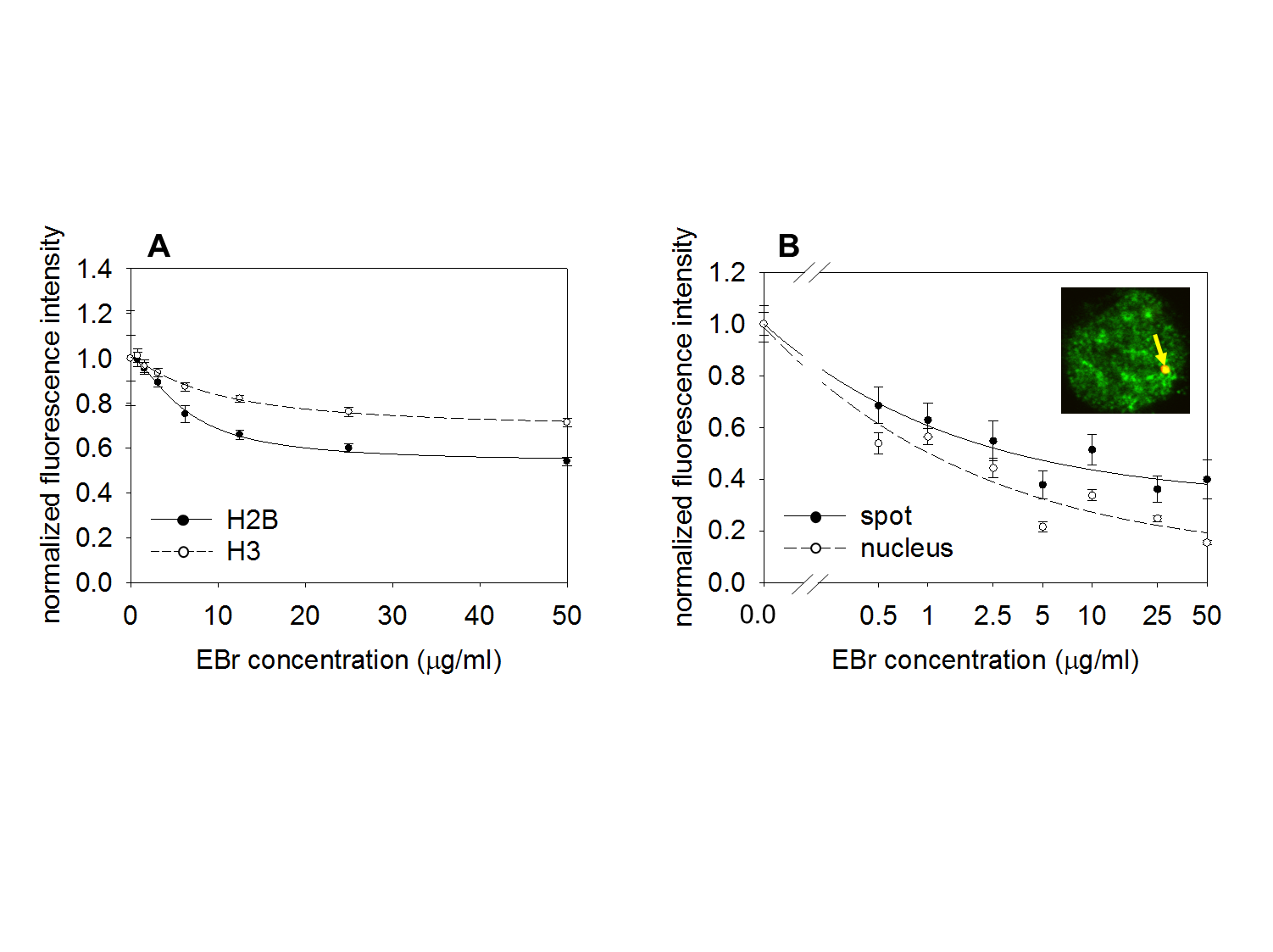


Supplementary Fig. S5

Measurements using alternative instrumental platforms.

(A) EBr elution of H2B-GFP and H3-GFP in the presence of 0.25 M salt, measured by flow-cytometry. Curves refer to G1 phase cells gated according to their DNA fluorescence intensity distribution and the error bars represent SEM of ~5000 G1 nuclei.

(B) EBr elution of H3K4me3 measured in a genome integrated chromatin domain. Elution profiles measured by CLSM in a nuclear domain visualized by the LacO array-bound mCherry-Lac-repressor-ASH2L (—●—) and in the whole nucleus (– – ○ – –). The inset shows H3K4me3 immunofluorescence (green) overlayed with the mCherry fluorescence signal of LacR (red). Error bars represent SEM of 15 nuclei measured by CLSM.


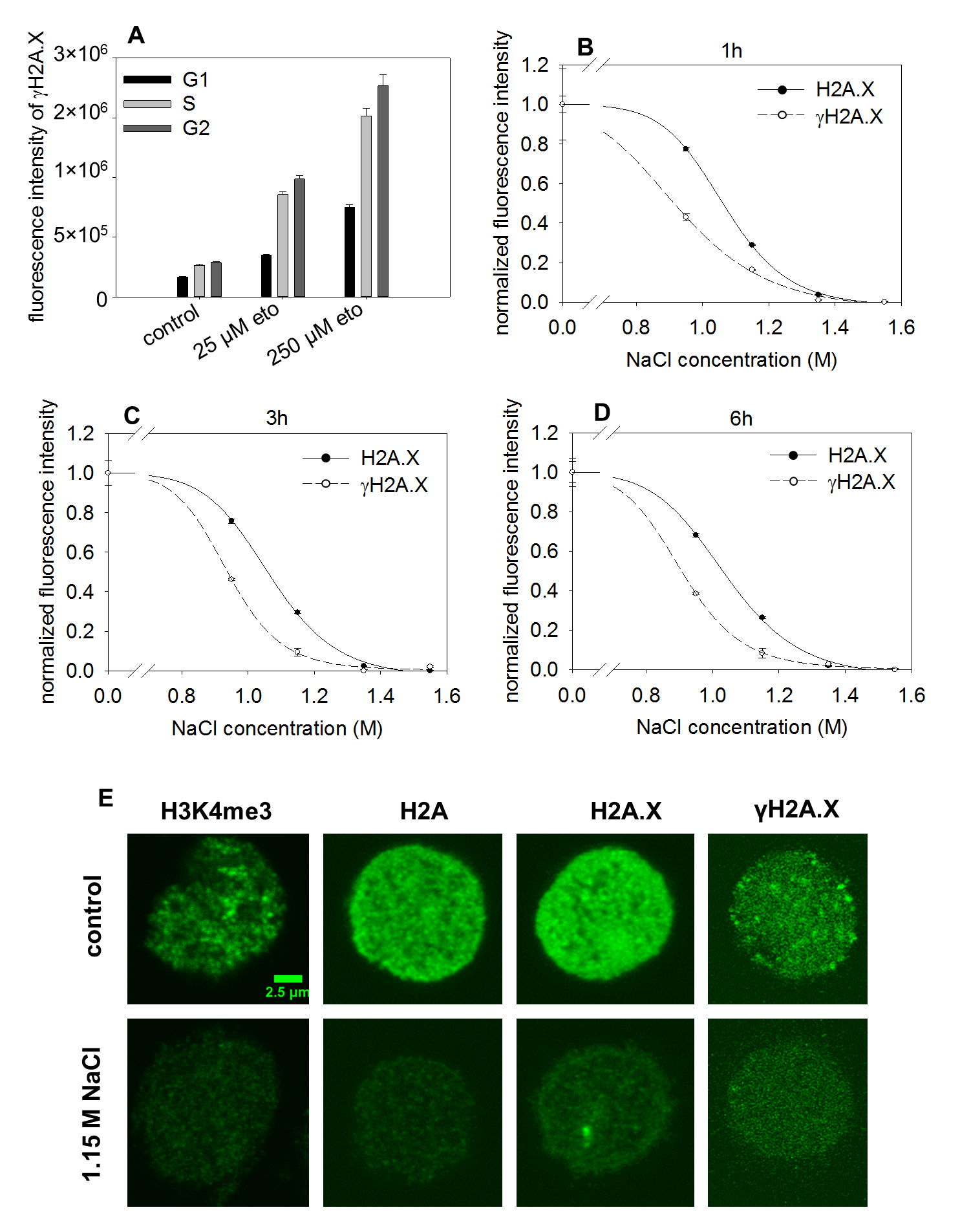


Supplementary Fig. S6

H3K4me3, H2A, H2A.X and γH2A.X localization, expression and stability features.

(A) LSC evaluation of nuclear γH2A.X immunofluorescence intensities after exposure of HCT116 cells to different concentrations of etoposide, for 1 h. Mean fluorescence intensities in the different cell cycle phases gated according to their DNA fluorescence distribution are shown. Error bars represent SEM of ~600 G1 nuclei measured by LSC.

(B-D) Salt elution curves of H2A.X and γH2A.X recorded after 1 h (C), 3 h (D) and 6 h (E) treatment of the cells with 25 µM etoposide. γH2A.X was labeled together in the same sample with either H2A or H2A.X, using species-specific secondary antibodies.

(E) Nuclear localization of H3K4me3, H2A, H2A.X and γH2A.X. CLSM immunofluorescence images of HCT116 nuclei, with or without salt treatment.

The curves refer to G1 phase cells gated according to their DNA fluorescence distribution. Error bars represent SEM of ~600 G1 nuclei measured by LSC.


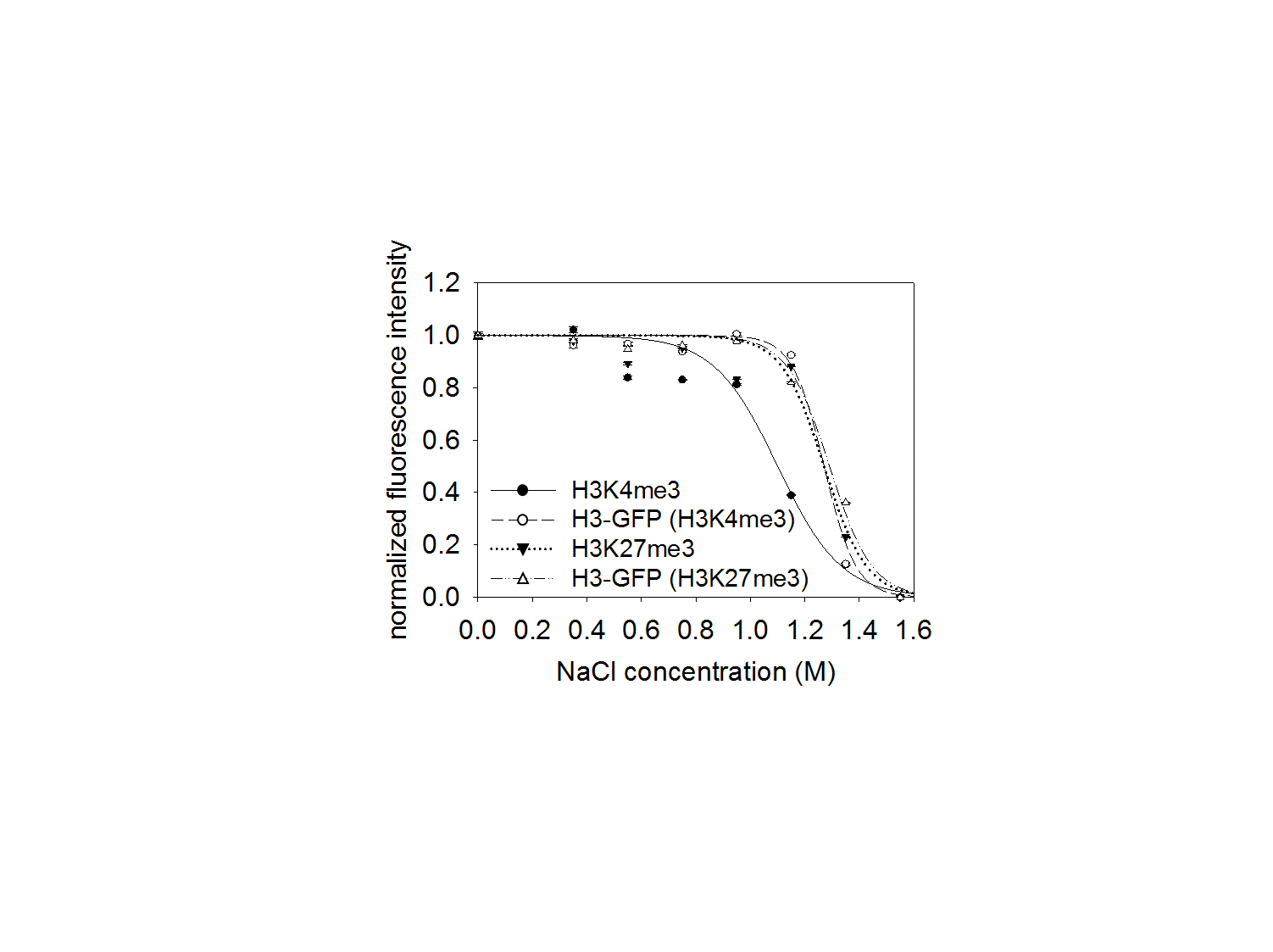


Supplementary Fig. S7

Internal controls of the H3K4me3/H3K27me3 salt elution measurements in Fig. 3C.

H3K4me3 - H3-GFP and H3K27me3 - H3-GFP elution curves, plotting the PTM data of Fig. 3C together with the H3-GFP signals. The two PTMs were measured in separate samples of H3-GFP HeLa nuclei, with the GFP signal recorded as internal control. Error bars represent SEM of ~600 G1 nuclei measured by LSC.


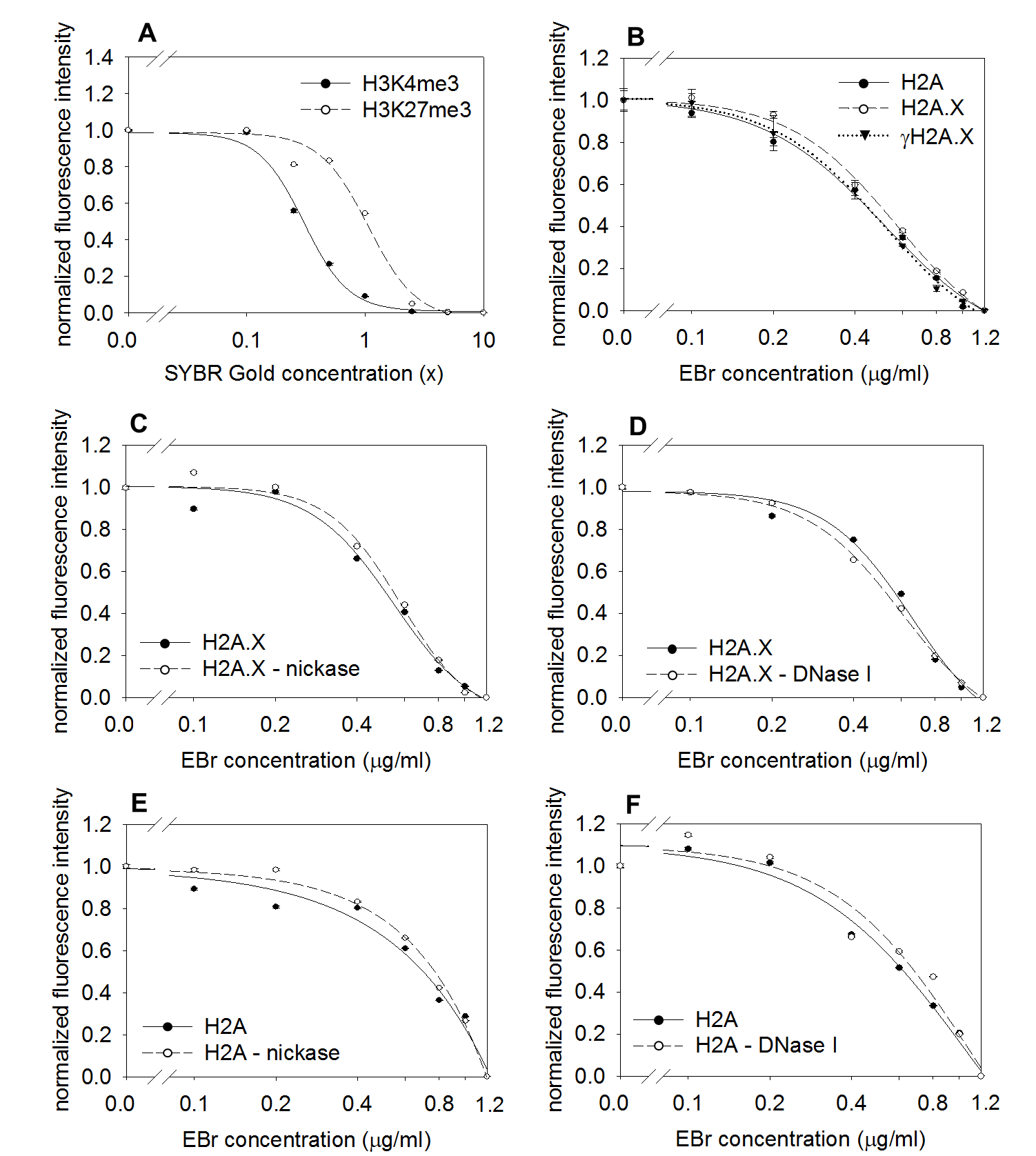


Supplementary Fig. S8

H3K4me3 SYBR GOLD elution in HeLa nuclei (A), and demonstration of the lack of effect of nicking on γH2A.X, H2A.X and H2A EBr elution profiles (B-F).

(A) The experiment of Fig. 4B reproduced using HeLa cells.

(B) EBr elution profiles of γH2A.X, H2A.X and H2A, in the nuclei of etoposide treated HCT116 cells.

(C and D) EBr elution curves of H2A.X after nickase (C) or DNase I (D) treatment of the nuclei (compare with Fig. 4C, D).

(E and F) EBr elution curves of H2A after nickase (E) or DNase I (F) treatment of the nuclei (compare with Fig. 4E and F).

The concentration of the intercalators are shown in a logarithmic scale.The curves refer to G1 phase cells gated according to their DNA fluorescence distribution. Error bars represent SEM of ~600 G1 nuclei measured by LSC.


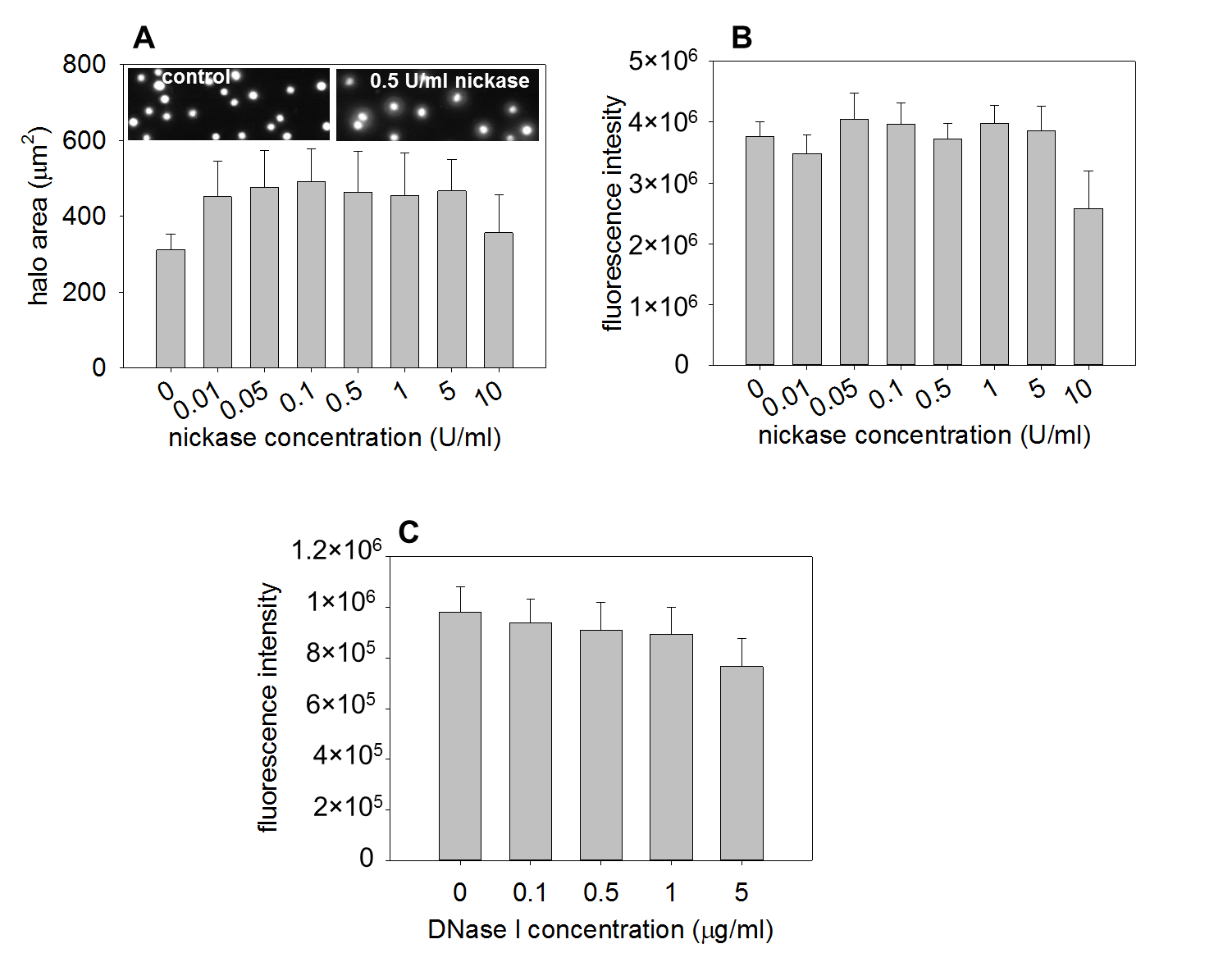


Supplementary Fig. S9

Optimization of nuclease treatment conditions.

(A and B) Halo size (A) and the amount of DNA remaining in the nuclei (B; based on EBr fluorescence) after digestion with nickase used at different concentrations.

(C) The amount of DNA remaining in the nuclei after digestion with DNase I used at different concentrations.

The bar charts refer to G1 phase cells gated according to their DNA fluorescence distribution. Error bars represent SD of ~600 G1 nuclei measured by LSC.


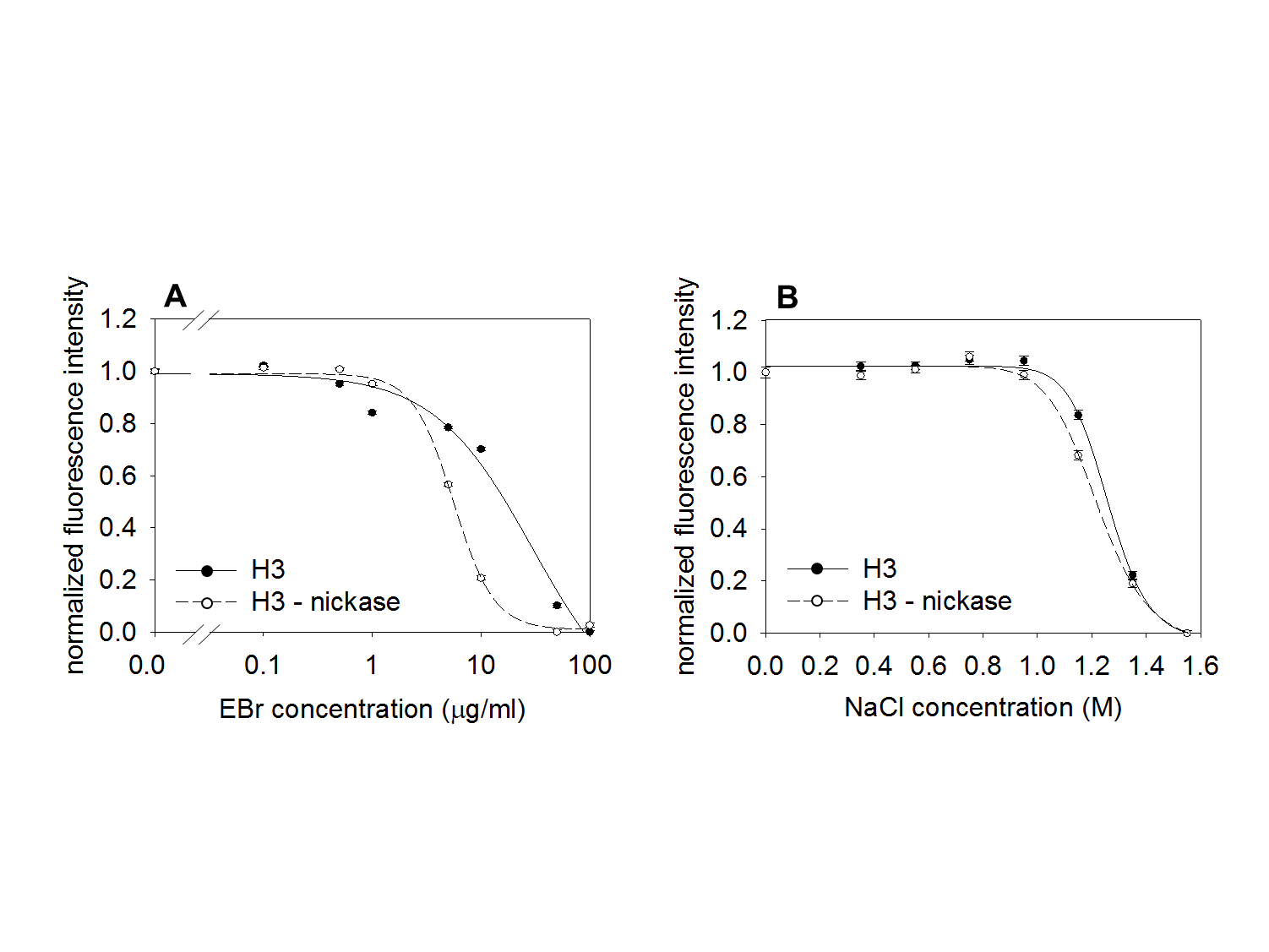


Supplementary Fig. S10

Effects of DNA relaxation elicited by nucleases on (H3-H4)_2_ stability.

(A and B) Elution curves of H3 after nickase treatment of the nuclei, measured by EBr (A) or salt elution (B). The concentration of the intercalators are shown in a logarithmic scale in panel A. The curves refer to G1 phase H3-GFP cells gated according to their DNA fluorescence distribution. Error bars represent SEM of ~600 G1 nuclei measured by LSC.


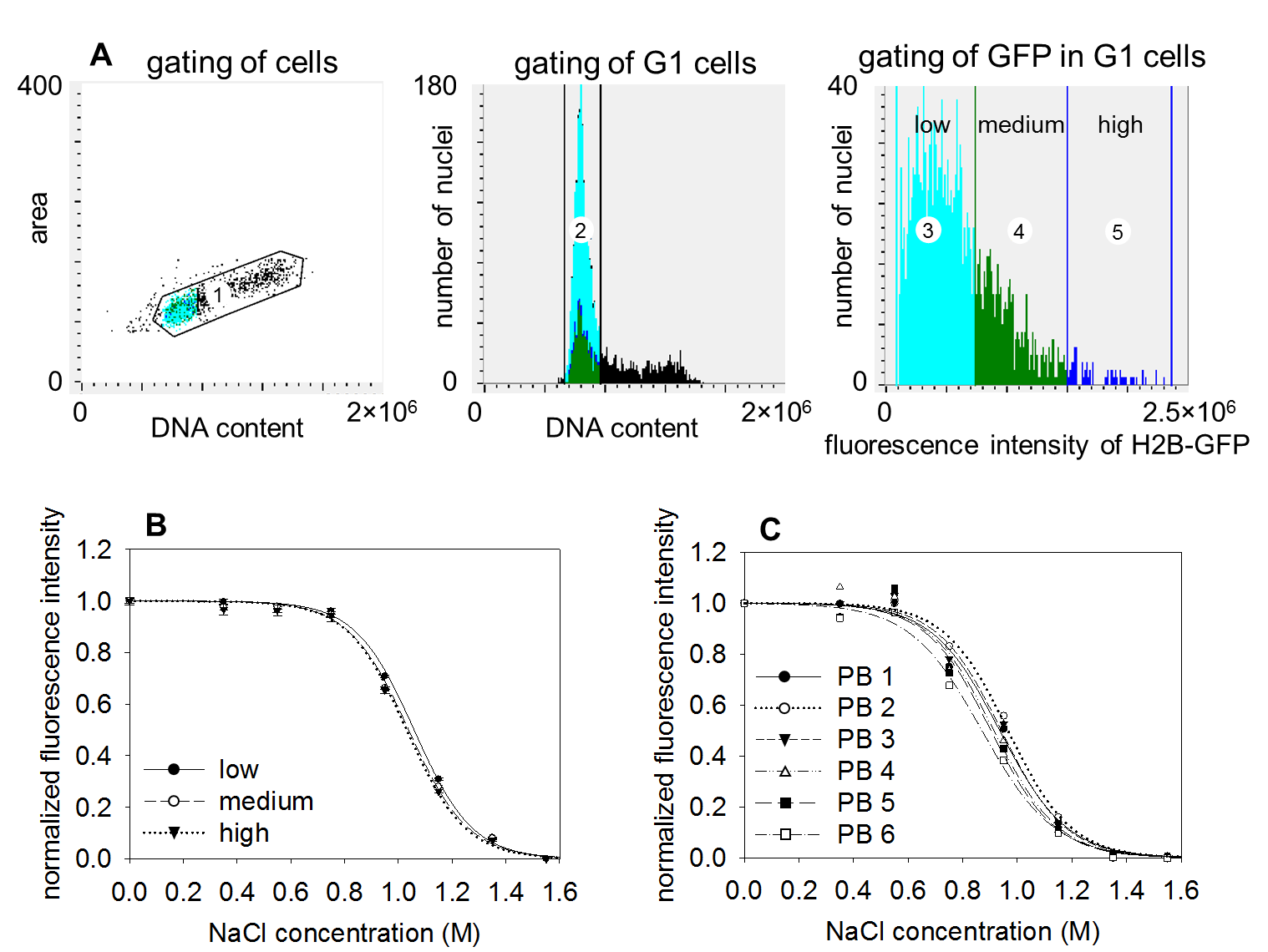


Supplementary Fig. S11

LSC analyses of the relationship between stability and expression levels of GFP-tagged H2B histones in HeLa cell nuclei and H2B NaCl elution experiments using different permeabilization buffers.

(A) Gating protocol. Nuclei within the primary gate (gate 1; left) were further gated (gate 2; middle) to select G1 nuclei. Elution curves obtained for the nuclei within Gates 3, 4 and 5 on the GFP intensity distribution histogram (right) were compared.

(B) Salt elution curves of low, medium and high H2B-GFP expressor cells. Nuclei in each well were measured twice, before and after elution using a particular eluent concentration.

(C) H2B salt elution curves measured by LSC, using different permeabilization buffers. PB1: 1% (v/v) Triton X-100 dissolved in 1×PBS/EDTA; PB2: 0.1% (v/v) Triton X-100 dissolved in 1×PBS/EDTA; PB3: 0.4% (v/v) Triton X-100 and 0.3 M NaCl dissolved in Tris-EDTA; PB4: 0.5% (v/v) Tween-20 dissolved in 1×PBS/EDTA; PB5: 1% NP-40 (v/v) dissolved in 1×PBS/EDTA; PB6: 1 mg/ml Saponin dissolved in „physiological buffer” containing 100 mg/ml BSA according to ref.[^4^](#_ENREF_4).

The curves refer to G1 phase cells gated according to the DNA fluorescence distributions. Error bars represent SEM of ~600 G1 nuclei measured by LSC.


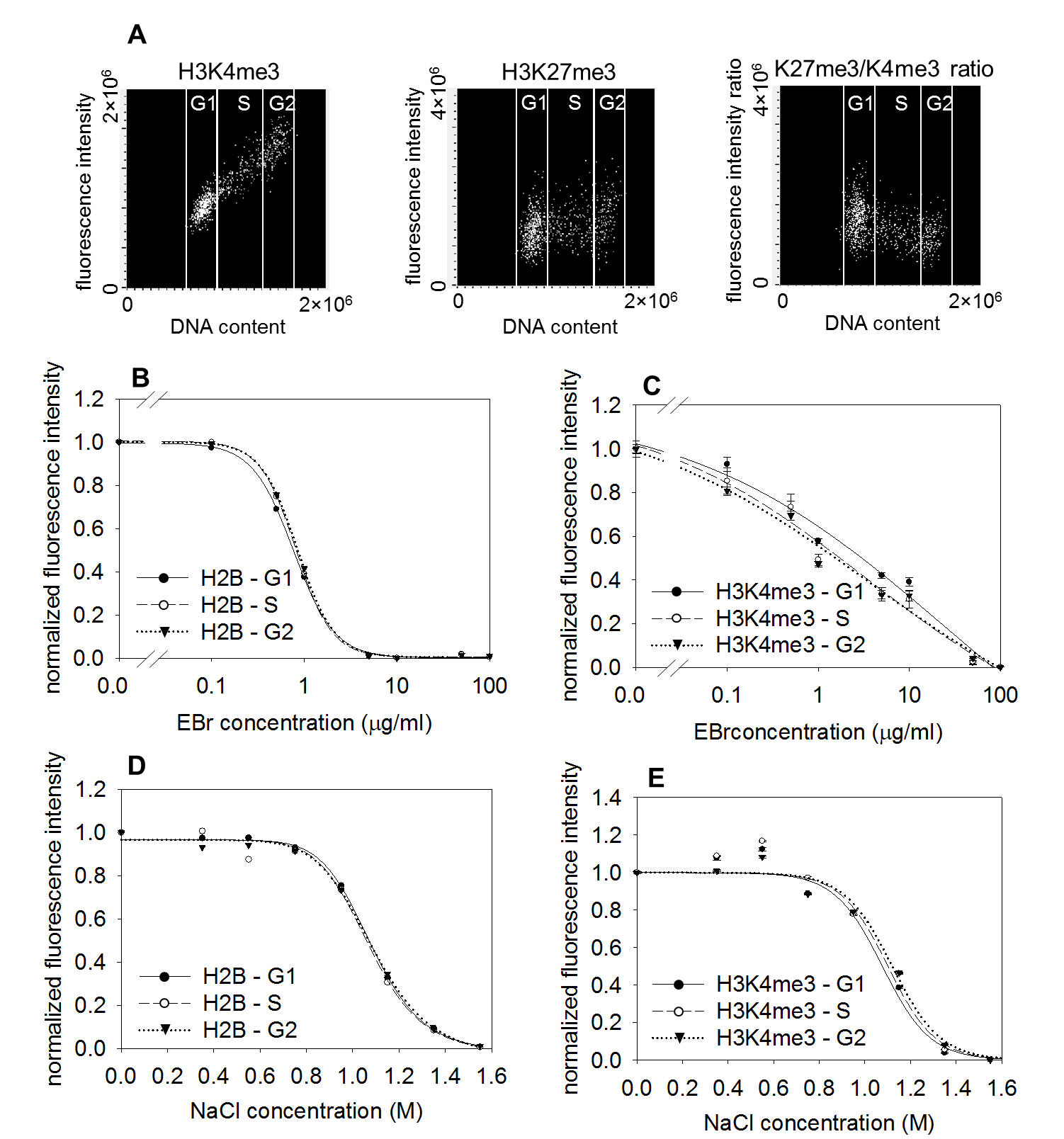


Supplementary Fig. S12

Cell-cycle resolution of nucleosome stability features.

(A) Immunofluorescence – DNA content scattergrams: H3K4me3 (left panel), H3K27me3 (middle), H3K27me3/K4me3 ratio (right). Each dot on the scattergrams represents a single nucleus co-labeled with mouse monoclonal anti-H3K4me3 and rabbit monoclonal anti-H3K27me3, stained by the appropriate dye-conjugated secondary antibodies.

(B-E) Comparison of nucleosome stability in the different phases of the cell-cycle. EBr elution profiles of H2B-GFP (B), H3K4me3 (C), and NaCl elution profiles of H2B-GFP (D), H3K4me3 (E) in the nuclei of G1, S and G2 phase, histone-GFP expressor HeLa cells identified by gating on the DNA fluorescence distributions within non-synchronized cell populations. (See also: Fig. 2A,C; Fig. 3A,C)

The concentration of the intercalators are shown in a logarithmic scale in panels B, C. The curves refer to G1 phase cells gated according to the DNA fluorescence distributions. Error bars represent SEM of ~600 G1 nuclei measured by LSC.


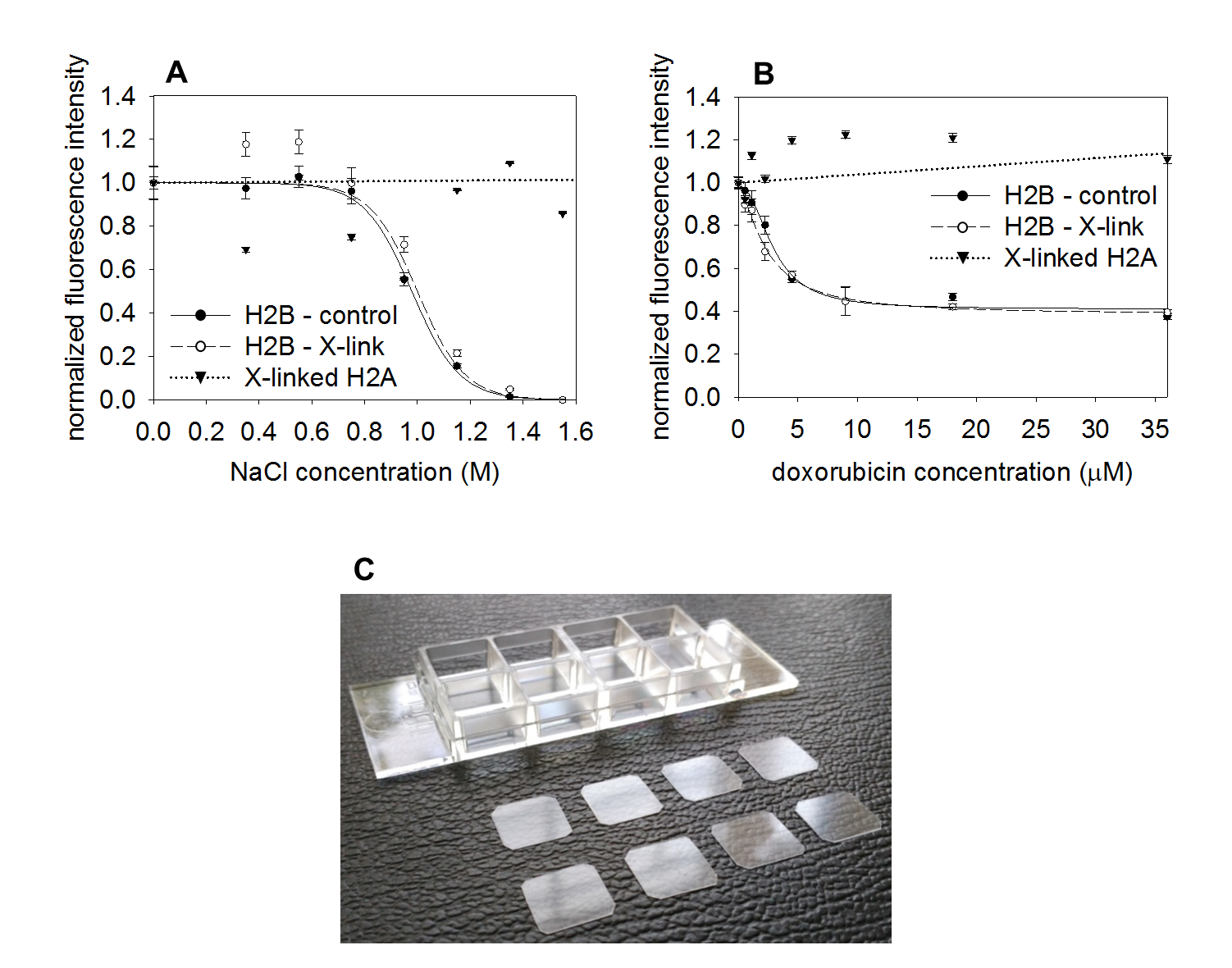


Supplementary Fig. S13

Effect of antibody cross-linking before elution.

(A and B) Effect of H2A immuno-cross-linking (X-link) by bivalent antibodies on the salt (A) and intercalator (B) elution profiles of the cross-linked histone and on its dimerization partner, measured in histone-GFP expressor HeLa nuclei. Control: samples with no cross-linking. For antibody X-linking, labeling of histones with primary and secondary antibodies was performed prior to salt or intercalator treatment.

The curves refer to G1 phase cells gated according to the DNA fluorescence distributions. Error bars represent SEM of ~600 G1 nuclei measured by LSC.

(C) Plastic coverslips used to prevent meniscus formation and drying out of the agarose-embedded cells in the wells of the 8-well Ibidi cell culture chambers.

| **treatment** | **labeling** | **cell to cell C.V.** | **sample to sample C.V.** |
| --- | --- | --- | --- |
| EBr | H3-GFP | 0.51 | 0.25 |
|  | H2B-GFP | 0.75 | 0.13 |
|  | H3K4me3 IgG | 0.24 | 0.11 |
|  | H3K27me3 IgG | 0.36 | 0.25 |
|  | K27me3/K4me3 | 0.25 | ─ |
|  | H3K4me3 Fab | 0.31 | ─ |
| NaCl | H3-GFP | 0.41 | 0.15 |
|  | H2B-GFP | 0.94 | 0.16 |
|  | H3K4me3 IgG | 0.29 | 0.15 |
|  | H3K27me3 IgG | 0.49 | 0.16 |
|  | K27me3/K4me3 | 0.24 | ─ |

Supplementary Table S1

Coefficients of variation of elution measurements.

Sample-to-sample coefficient of variation (C.V.= S.D./mean) values were calculated for the distribution of IgG labeled H3K4me3 and H3K27me3, Fab labeled H3K4me3 and of H2B-GFP or H3-GFP based on 3 independent LSC elution experiments performed on HeLa cells. The cell-to-cell C.V. values, from top to bottom in the Table, were calculated for the distribution histograms shown in Fig. 2A (H2B and H3 on ice), Fig. 2C (H3K4me3 and H3K27 me3), Fig. 3A (H2B and H3), Fig. 3C (H3K4me3 and H3K27 me3), respectively. The H3K27me3/H3K4me3 ratios were calculated from elution curves of co-labeled (K4me3-Alexa488, K27me3-Alexa647) HeLa nuclei from the measured H3K4me3 data point closest to the EC50. H3K4me3 Fab elution experiment: elution curve not shown. Regarding calculations see also Supplementary Methods.

**SUPPLEMENTARY METHODS**

**Immunofluorescence labeling with the TAF3 PHD domain**

Agarose embedded and permeabilized HeLa nuclei were incubated with 500 μl 5% (m/v) Blotto Non-Fat Dry Milk in 1×PBS/EDTA for 30 minutes on ice, to decrease nonspecific staining. The samples were washed with 500 μl ice cold 1×PBS/EDTA three times and indirect immunofluorescence labeling was performed using the GST-tagged PHD domain of the reader protein TAF3[^5^](#_ENREF_5) diluted in 150 μl of 1×PBS/EDTA/1% BSA at 4°C, overnight. Prior to labeling, TAF3 was filtered through a Pierce Concentrator 150K MWCO (Thermo Fisher Scientific, Waltham, Massachusetts, USA) to remove aggregates. After filtration, the concentration of the stock solution was 0.2 mg/ml which was applied to the wells at a titer of 1:400. After labeling with TAF3, the nuclei were washed with 500 μl ice cold 1×PBS/EDTA three times for 10 minutes and the samples were treated with EBr or doxorubicin intercalators as described above. Following treatment, labeling with the secondary antibody was performed in 150 μl 1×PBS/EDTA for two hours on ice, using A488 conjugated rabbit polyconal anti-GST antibody (Thermo Fisher Scientific, Waltham, Massachusetts, USA). The secondary antibody was used at a titer of 1:800, diluted in 1×PBS/EDTA from its 2 mg/ml stock solution. After labeling with the secondary antibody, nuclei were washed with 500 μl ice cold 1×PBS/EDTA three times. Then the samples were fixed with formaldehyde, stained with propidium–iodide and the fluorescence intensity distributions were recorded by LSC as described above.

**Flow cytometry**

Flow cytometric measurements were performed using a Becton Dickinson FACScan instrument (San Jose, California, USA). GFP and PI were excited by a 488 nm Argon ion laser, the emitted fluorescence signals were detected via a 520 nm (FL1) and a 575 nm (FL2) filter, respectively. Fluorescence signals were collected in the logarithmic mode and the data were analyzed using the ReFlex software[^6^](#_ENREF_6).

**Nucleosome stability assay performed in a stably integrated genetic locus**

U2OS cells containing a LacO-I-SceI-TetO array were transiently transfected with mCherry-LacR-ASH2L, a tagged Lac-repressor construct fused with the histone methyl transferase (ASH2L) gene[^7^](#_ENREF_7). Transfection was performed by the Fugene transfection reagent (Promega, Madison, Wisconsin, USA), following the manufacturer’s instructions. Agarose- embedded and permeabilized, transfected U2OS cells were incubated with 500 μl 5% (m/v) Blotto Non-Fat Dry Milk in 1×PBS/EDTA for 30 minutes on ice, to decrease nonspecific staining. The blocking solution was washed out with 500 μl ice cold 1×PBS/EDTA three times and indirect immunofluorescence labeling was performed using rat monoclonal A488-conjugated anti-mCherry antibody (Thermo Fisher Scientific, Waltham, Massachusetts, USA) diluted at a titer of 1:800 in 150 μl of 1×PBS/EDTA/1% BSA, at 4°C, overnight, to better visualize, as well as to cross-link the mCherry-Lac-repressor-ASH2L fusion protein and thereby prevent its dissociation during the elution experiment. After labeling, the nuclei were washed with 500 μl ice cold 1×PBS/EDTA three times and exposed to EBr as described above. After treatment, indirect immunofluorescence labeling of H3K4me3 nucleosomes was performed using unlabeled primary and A647-conjugated goat anti-mouse secondary antibody. The fluorescence signal of H3K4me3 was measured in the LacO spot and in the whole nucleus by CLSM.

**Confocal Laser Scanning Microscopy (CLSM)**

Confocal images were taken using an FLUOVIEW FV 1000 confocal microscope (Olympus, Center Valley, Pennsylvania, USA) based on an inverted IX-81 stand with an UPLS APO 60× (NA 1.35) oil immersion objective. GFP and A488 were excited by a 488 nm Argon ion laser. A647 and PI were excited by a 633 nm and 543 nm HeNe laser, respectively. Image analysis was performed using the Image J software (http://imagej.nih.gov/ij/).

**Immuno-cross-linking experiments**

Agarose embedded and permeabilized HeLa cells expressing H2B-GFP were incubated with 500 μl 5% (m/v) Blotto Non-Fat Dry Milk dissolved in 1×PBS/EDTA, for 30 minutes, on ice, to decrease nonspecific binding. The blocking solution was washed out with 500 μl ice cold 1×PBS/EDTA three times and indirect immunofluorescence labeling was performed using rabbit polyclonal anti-H2A antibody at a titer of 1:800, diluted in 150 μl of 1×PBS/EDTA/1% BSA, at 4°C, overnight. After labeling with the anti-H2A antibody, the nuclei were washed with 500 μl ice cold 1×PBS/EDTA three times. Anti-H2A labeled histones were cross-linked with the secondary antibody in 150 μl 1×PBS/EDTA for two hours on ice, using A647-conjugated goat anti-rabbit IgG. The secondary antibody was used at a titer of 1:800, diluted in 1×PBS/EDTA from its 2 mg/ml stock solution. After labeling with the secondary antibody, nuclei were washed with 500 μl ice cold 1×PBS/EDTA three times and the samples were treated with NaCl or doxorubicin solutions as described above. Then the samples were fixed with formaldehyde, stained with propidium iodide and the fluorescence intensity distributions were recorded by LSC, as described above.

**Calculation of coefficients of variation**

Sample-to-sample coefficient of variation values (Supplementary Table S1) were calculated from the formula: C.V.= S.D./mean, where standard deviations were calculated from the means of three independent experiments. To calculate the cell-to-cell C.V. values, standard deviations of the fluorescence intensity distributions of cell populations analyzed in the given experiment were used. The measured points closest to EC_50_ on the normalized elution curves were used as the mean values.

**SUPPLEMENTARY DISCUSSION**

H2A.X differs from H2A by its C-terminus containing the serine residue (position 139 in humans), which rapidly becomes phosphorylated upon double-strand break (DSB) induction. Human H2A and H2A.X also differ by four amino acids including a residue (N39H) located in the interfacial region of the H2A–H2B dimers. γH2A.X is widely known as a DNA repair module and it is extensively used to monitor DNA damage response (DDR)[^10^](#_ENREF_10). In mammals, many hundreds of histone H2A.X molecules become phosphorylated (in ‘γH2A.X foci’) within 10–30 min in the chromatin region flanking each newly formed DSB (up to several Mbs;. ATM-dependent H2A.X phosphorylation occurs also in the absence of genotoxic stimuli and appears to play a role in HMGA2-induced transcriptional activation[^13^](#_ENREF_13). Endogenous γH2A.X was shown to be enriched in the transcription start site (TSS) of actively transcribed genes[^14^](#_ENREF_14). Both H2A.X and γH2A.X are readily evicted by the anthracyclin drugs doxorubicin and daunorubicin, an effect attributed to DNA intercalation rather than to the double-strand breaks (DSBs) induced via the well-known topoisomerase II inhibitory activity of these chemotherapeutic agents[^15^](#_ENREF_15).

QINESIn is a very promising analytical platform with high-throughput screening potential for chemical profiling of the genome in search of intercalators with PTM-specific effects[^16^](#_ENREF_16). It may also be a method of choice for studying the mechanisms of action of anthracycline drugs, which presumably involve torsion-induced nucleosome destabilization. It may also be a useful tool for studying the influence of chromatin remodelling complexes and histone chaperones on nucleosome disassembly in different model organisms including plant cells[^19^](#_ENREF_19). It may complement genomic approaches in the analyses of regulated histone turnover[^20^](#_ENREF_20),[^21^](#_ENREF_21). The functional consequences of cell-cycle dependent epigenetic changes could also be monitored through the spectacles of QINESIn. PTMs located within the core histone fold are particularly likely to have a direct influence on nucleosome stability and could be readily investigated using this platform. Considering its sensitivity to changes in the supercoiled state of DNA, the effects of topoisomerase inhibitors could also be conveniently studied using the method. The assay can be implemented using other chromatin destabilizing agents such as urea[^24^](#_ENREF_24). Research areas where the application of QINESIn is expected to provide new insights include examples such as: "scheduled DNA damage/repair” at gene promoters[^25^](#_ENREF_25) or during pause release and elongation[^26^](#_ENREF_26), addressing the effect of H1 and its variants on nucleosome stability features arising from core histones in experiments where linker histone release is prevented by antibody crosslinking (like in Supplementary Fig. S13A, B), or assessing the role of nonhistone protein binding in influencing nucleosome stability features, among others.

**SUPPLEMENTARY REFERENCES**

1 Daniel, B., Balint, B. L., Nagy, Z. S. & Nagy, L. Mapping the genomic binding sites of the activated retinoid X receptor in murine bone marrow-derived macrophages using chromatin immunoprecipitation sequencing. *Methods in molecular biology* **1204**, 15-24, doi:10.1007/978-1-4939-1346-6_2 (2014).

2 Kimura, H., Hayashi-Takanaka, Y., Goto, Y., Takizawa, N. & Nozaki, N. The organization of histone H3 modifications as revealed by a panel of specific monoclonal antibodies. *Cell structure and function* **33**, 61-73 (2008).

3 Hayashi-Takanaka, Y. *et al.* Tracking epigenetic histone modifications in single cells using Fab-based live endogenous modification labeling. *Nucleic Acids Res* **39**, 6475-6488, doi:10.1093/nar/gkr343 (2011).

4 Pombo, A. *et al.* Regional specialization in human nuclei: visualization of discrete sites of transcription by RNA polymerase III. *The EMBO journal* **18**, 2241-2253, doi:10.1093/emboj/18.8.2241 (1999).

5 Kungulovski, G. *et al.* Application of histone modification-specific interaction domains as an alternative to antibodies. *Genome research* **24**, 1842-1853, doi:10.1101/gr.170985.113 (2014).

6 Szentesi, G. *et al.* Computer program for determining fluorescence resonance energy transfer efficiency from flow cytometric data on a cell-by-cell basis. *Computer methods and programs in biomedicine* **75**, 201-211, doi:10.1016/j.cmpb.2004.02.004 (2004).

7 Burman, B., Zhang, Z. Z., Pegoraro, G., Lieb, J. D. & Misteli, T. Histone modifications predispose genome regions to breakage and translocation. *Genes & development* **29**, 1393-1402, doi:10.1101/gad.262170.115 (2015).

8 Bonisch, C. & Hake, S. B. Histone H2A variants in nucleosomes and chromatin: more or less stable? *Nucleic Acids Res* **40**, 10719-10741, doi:10.1093/nar/gks865 (2012).

9 Fink, M., Imholz, D. & Thoma, F. Contribution of the serine 129 of histone H2A to chromatin structure. *Molecular and cellular biology* **27**, 3589-3600, doi:10.1128/MCB.02077-06 (2007).

10 Bonner, W. M. *et al.* GammaH2AX and cancer. *Nature reviews. Cancer* **8**, 957-967, doi:10.1038/nrc2523 (2008).

11 Rogakou, E. P., Boon, C., Redon, C. & Bonner, W. M. Megabase chromatin domains involved in DNA double-strand breaks in vivo. *J Cell Biol* **146**, 905-916 (1999).

12 Redon, C. E. *et al.* gamma-H2AX and other histone post-translational modifications in the clinic. *Biochimica et biophysica acta* **1819**, 743-756, doi:10.1016/j.bbagrm.2012.02.021 (2012).

13 Singh, I. *et al.* High mobility group protein-mediated transcription requires DNA damage marker gamma-H2AX. *Cell research* **25**, 837-850, doi:10.1038/cr.2015.67 (2015).

14 Seo, J. *et al.* Genome-wide profiles of H2AX and gamma-H2AX differentiate endogenous and exogenous DNA damage hotspots in human cells. *Nucleic Acids Res* **40**, 5965-5974, doi:10.1093/nar/gks287 (2012).

15 Pang, B. *et al.* Drug-induced histone eviction from open chromatin contributes to the chemotherapeutic effects of doxorubicin. *Nature communications* **4**, 1908, doi:10.1038/ncomms2921 (2013).

16 Pang, B., de Jong, J., Qiao, X., Wessels, L. F. & Neefjes, J. Chemical profiling of the genome with anti-cancer drugs defines target specificities. *Nature chemical biology* **11**, 472-480, doi:10.1038/nchembio.1811 (2015).

17 Yang, F., Teves, S. S., Kemp, C. J. & Henikoff, S. Doxorubicin, DNA torsion, and chromatin dynamics. *Biochimica et biophysica acta* **1845**, 84-89, doi:10.1016/j.bbcan.2013.12.002 (2014).

18 Rabbani, A., Iskandar, M. & Ausio, J. Daunomycin-induced unfolding and aggregation of chromatin. *The Journal of biological chemistry* **274**, 18401-18406 (1999).

19 Strenkert, D., Schmollinger, S., Sommer, F., Schulz-Raffelt, M. & Schroda, M. Transcription factor-dependent chromatin remodeling at heat shock and copper-responsive promoters in Chlamydomonas reinhardtii. *The Plant cell* **23**, 2285-2301, doi:10.1105/tpc.111.085266 (2011).

20 Zentner, G. E. & Henikoff, S. Regulation of nucleosome dynamics by histone modifications. *Nature structural & molecular biology* **20**, 259-266, doi:10.1038/nsmb.2470 (2013).

21 Teves, S. S., Deal, R. B. & Henikoff, S. Measuring genome-wide nucleosome turnover using CATCH-IT. *Methods in enzymology* **513**, 169-184, doi:10.1016/B978-0-12-391938-0.00007-0 (2012).

22 Kebede, A. F., Schneider, R. & Daujat, S. Novel types and sites of histone modifications emerge as players in the transcriptional regulation contest. *The FEBS journal* **282**, 1658-1674, doi:10.1111/febs.13047 (2015).

23 Pradeepa, M. M. *et al.* Histone H3 globular domain acetylation identifies a new class of enhancers. *Nature genetics* **48**, 681-686, doi:10.1038/ng.3550 (2016).

24 Hegedus, E., Kokai, E., Kotlyar, A., Dombradi, V. & Szabo, G. Separation of 1-23-kb complementary DNA strands by urea-agarose gel electrophoresis. *Nucleic Acids Res* **37**, e112, doi:10.1093/nar/gkp539 (2009).

25 Fong, Y. W., Cattoglio, C. & Tjian, R. The intertwined roles of transcription and repair proteins. *Molecular cell* **52**, 291-302, doi:10.1016/j.molcel.2013.10.018 (2013).

26 Bunch, H. *et al.* Transcriptional elongation requires DNA break-induced signalling. *Nat Commun* **6**, 10191, doi:10.1038/ncomms10191 (2015).

27 Thoma, F., Koller, T. & Klug, A. Involvement of histone H1 in the organization of the nucleosome and of the salt-dependent superstructures of chromatin. *J Cell Biol* **83**, 403-427 (1979).

28 Kalashnikova, A. A., Rogge, R. A. & Hansen, J. C. Linker histone H1 and protein-protein interactions. *Biochimica et biophysica acta* **1859**, 455-461, doi:10.1016/j.bbagrm.2015.10.004 (2016).

29 Schroter, H., Maier, G., Ponstingl, H. & Nordheim, A. DNA intercalators induce specific release of HMG 14, HMG 17 and other DNA-binding proteins from chicken erythrocyte chromatin. *The EMBO journal* **4**, 3867-3872 (1985).

30 Hamana, K. & Kawada, K. Release of nucleosomes from nuclei by bleomycin-induced DNA strand scission. *Biochemistry international* **18**, 971-979 (1989).
